# Machine learning applications for thermochemical and kinetic property prediction

**DOI:** 10.1515/revce-2024-0027

**Published:** 2024-11-29

**Authors:** Lowie Tomme, Yannick Ureel, Maarten R. Dobbelaere, István Lengyel, Florence H. Vermeire, Christian V. Stevens, Kevin M. Van Geem

**Affiliations:** Laboratory for Chemical Technology, Department of Materials, Textiles and Chemical Engineering, 26656 Ghent University, Technologiepark 125, 9052 Gent, Belgium; ChemInsights LLC, Dover, DE 19901, USA; Department of Chemical Engineering, KU Leuven, Celestijnenlaan 200F, 3001 Leuven, Belgium; SynBioC Research Group, Department of Green Chemistry and Technology, Faculty of Bioscience Engineering, Ghent University, Ghent 9000, Belgium

**Keywords:** kinetic modeling, mechanism generation, artificial intelligence, thermodynamics, reaction rate

## Abstract

Detailed kinetic models play a crucial role in comprehending and enhancing chemical processes. A cornerstone of these models is accurate thermodynamic and kinetic properties, ensuring fundamental insights into the processes they describe. The prediction of these thermochemical and kinetic properties presents an opportunity for machine learning, given the challenges associated with their experimental or quantum chemical determination. This study reviews recent advancements in predicting thermochemical and kinetic properties for gas-phase, liquid-phase, and catalytic processes within kinetic modeling. We assess the state-of-the-art of machine learning in property prediction, focusing on three core aspects: data, representation, and model. Moreover, emphasis is placed on machine learning techniques to efficiently utilize available data, thereby enhancing model performance. Finally, we pinpoint the lack of high-quality data as a key obstacle in applying machine learning to detailed kinetic models. Accordingly, the generation of large new datasets and further development of data-efficient machine learning techniques are identified as pivotal steps in advancing machine learning’s role in kinetic modeling.

## 1 Introduction

**Figure 1:**
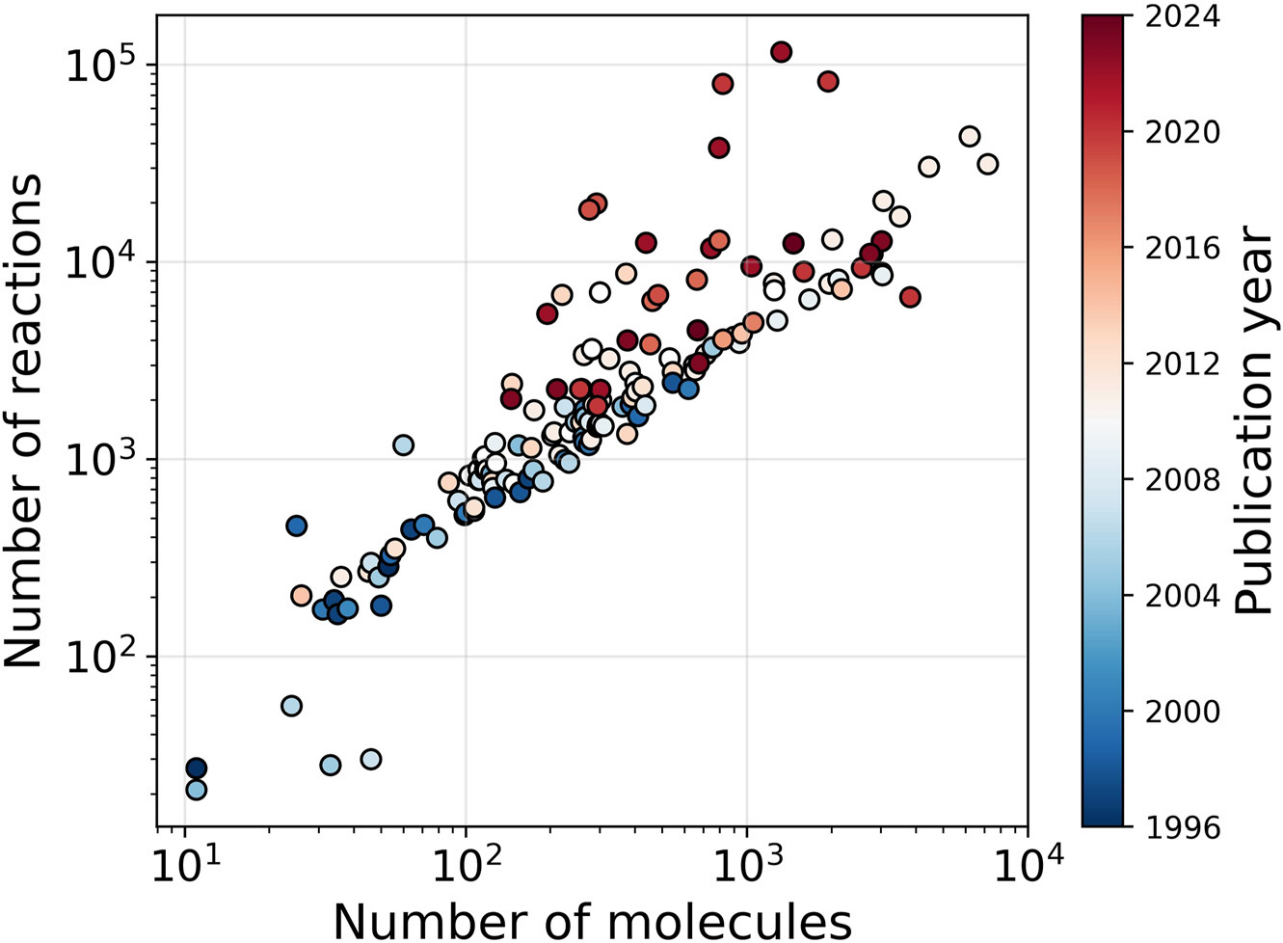
Evolution of the size of detailed kinetic gas-phase models from 1996 to 2024. Data until 2015 is based on the work of Van de Vijver et al. (2015).

Detailed kinetic models are an extremely powerful tool to gain insight into chemical processes. While experiments yield valuable data on process parameter effects, they often do not allow to gain mechanistic insights in a straightforward way. Detailed chemical kinetic models, on the other hand, provide insight into how the overall reaction proceeds but are tedious to develop. These detailed kinetic models consist of molecules, and reactions linking these molecules. For some processes, such as pyrolysis or combustion processes, these models can contain thousands of molecules and tens of thousands of reactions. Figure 1 shows the size of kinetic models of gas-phase processes developed during the last and previous decades, illustrating that the model size has increased over time.

Due to their size, large kinetic models are usually generated automatically. Over time, many groups have developed software for automatic kinetic model generation. Examples of such software tools include Genesys (Vandewiele et al. 2012), RMG (Gao et al. 2016), NETGEN (Broadbelt et al. 1994), MAMOX (Ranzi et al. 1997), and RING (Rangarajan et al. 2012). Automatic kinetic model generators typically operate based on user-defined reaction families. Initial molecules undergo reactions according to these families, producing new species. Subsequently, these newly formed species engage in further reactions via the specified families, resulting in a complex chemical reaction network. These automatically generated reaction networks, however, often need some manual manipulation, due to an incomplete reaction mechanism or an incorrect thermodynamic or kinetic parameter assignment (vide infra) (Faravelli et al. 2019). In practice, most detailed kinetic models are thus generated semi-automatically (Dogu et al. 2021; Miller et al. 2021; Zádor et al. 2011).

Describing the thermodynamics of the molecules and the kinetics of the reactions is essential for gaining insights into the processes these reaction networks model. While small kinetic models can have all their thermochemical and kinetic properties fitted to experimental data, this becomes impractical for larger models due to the vast number of parameters involved, risking overfitting. This can thus lead to different combinations of thermodynamic and kinetic parameters that can describe the experimental trends, due to a cancelation of errors (Katare et al. 2004; Park and Froment 1998). The obtained thermodynamic and kinetic values thus have a high uncertainty. Additionally, experimental data often only provide yields, output concentrations, and conversions, necessitating the selection of a reactor model alongside the kinetic model for regression purposes. For simple processes, a simple reactor model, which describes the concentration of the species as a function of time and the position in the reactor via simple mathematical equations, may be satisfactory. Examples of such simple reactor models include an ideal plug flow reactor model and a continuous stirred-tank reactor model. However, for more complex processes, constructing a suitable reactor model is more challenging (Xu and Froment 1989; Zapater et al. 2024). This increased complexity may introduce additional errors in parameter fitting, leading to wrong mechanistic insights.

**Figure 2:**
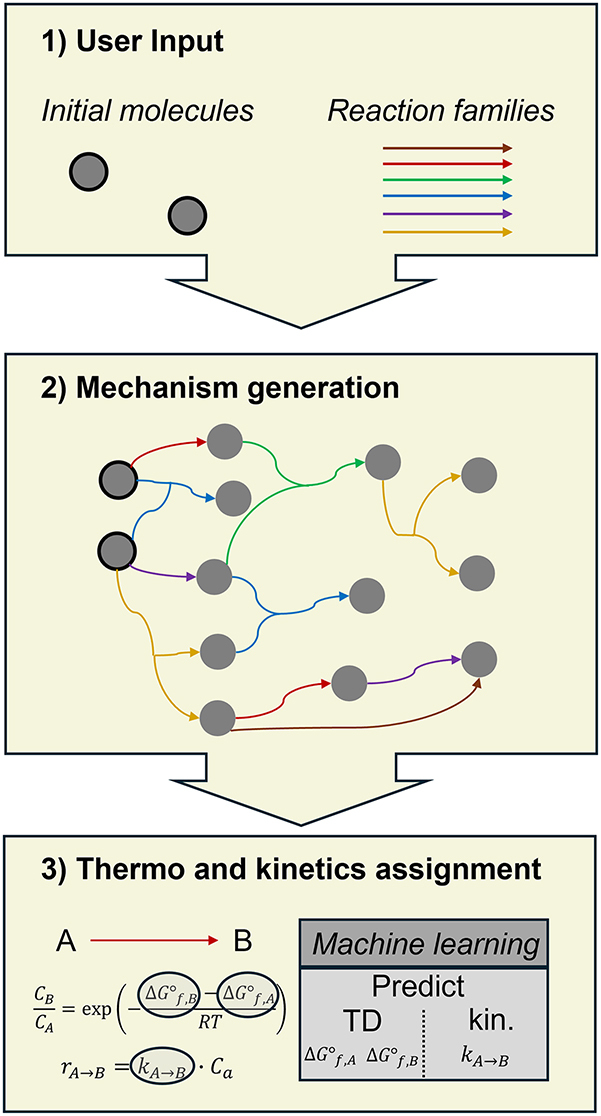
Process of automatic kinetic model generation and the role of machine learning in facilitating thermochemical and kinetic property prediction for this purpose.

Given these challenges, thermodynamics and kinetics are often computed using *in silico* methods, particularly for large kinetic models. While quantum chemical methods often offer accurate predictions, their computational demand is prohibitive for large mechanisms. Hence, less accurate but faster methods such as group additivity (Benson et al. 1969) and reaction rules are commonly employed. However, since quantum chemistry is time-consuming and faster methods sacrifice accuracy, there exists an opportunity for a more effective approach to calculate the necessary thermodynamic and kinetic properties. Machine learning emerges as a promising candidate to address this gap, given its demonstrated utility in various areas of chemical engineering such as computational fluid dynamics (CFD), rational fuel design (Fleitmann et al. 2023; Kuzhagaliyeva et al. 2022), and synthesis planning (Coley et al. 2018; Kochkov et al. 2021; Pirdashti et al. 2013). Machine learning has also been applied to predict outcomes of chemical processes. The input to the machine learning model is in this case process parameters such as inlet composition, temperature, and pressure. Similar to detailed kinetic models combined with a reactor model, the machine learning model predicts yields, conversions, and outlet concentrations. The usual purpose of these models is process optimization within a narrow range of process parameters. However, these machine learning models cannot be used to gain mechanistic insight into a process due to their “black box” nature. While the machine learning model may provide yield predictions, users lack insight into how these predictions are generated. Moreover, because these machine learning models are trained on experimental data, their performance beyond the training range may be uncertain. As mentioned above, machine learning can be used within a kinetic modeling approach, namely, to predict the thermodynamics and kinetics. The aforementioned method of automatic kinetic model generation, and where machine learning can be employed for property prediction is shown in Figure 2. This figure shows that first a reaction network is created based on the initial molecules and the reaction families. These families can include conventional reactions or more complex reaction types such well-skipping reactions, represented by the brown arrow. The latter reaction type, will, due to the complexity of its underlying physics, be addressed in future work. Once a reaction network is generated, the thermodynamic and kinetic parameters must be assigned. As presented in Figure 2, the thermodynamic property of interest is the Gibbs free reaction energy, determined by the enthalpy of formation, the intrinsic entropy, and the heat capacity of reactants and products. As these properties are temperature dependent, they are often represented by NASA polynomials, which allow to calculate the property value at a given temperature, as shown in equations (1)–(3). In these equations *h*_*i*_ represents the enthalpy of a species *i*, *s*_*i*_ its entropy, *C*_*p*,*i*_ its heat capacity, and *R* the gas constant.

(1) $$\frac{h_{i}}{R}=a_{1}T+\frac{a_{2}}{2}T^{2}+\frac{a_{3}}{3}T^{3}+\frac{a_{4}}{4}T^{4}+\frac{a_{5}}{5}T^{5}+a_{6}$$

(2) $$\frac{s_{i}}{R}=a_{1}\;\ln\;T+a_{2}T+\frac{a_{3}}{2}T^{2}+\frac{a_{4}}{3}T^{3}+\frac{a_{5}}{4}T^{4}+a_{7}$$

(3) $$\frac{C_{p,i}}{R}=a_{1}+a_{2}T+a_{3}T^{2}+a_{4}T^{3}+a_{5}T^{4}$$

When looking at liquid-phase processes, also solvation properties should be taken into account. Examples of such properties are the enthalpy of solvation Δ*H*_*s*olv_, or the Gibbs free energy of solvation Δ*G*_solv_. A third type of process is heterogeneous catalytic processes. For modeling these processes, the adsorption enthalpy and entropy are of great importance. Besides the thermodynamic effects, kinetics effects are also important in detailed kinetic models. The kinetics are described by the rate coefficients of the reactions in the models, as shown in Figure 2. The rate coefficients are often represented by the modified Arrhenius equation, presented in equation (4), in order to include temperature dependence. The pre-exponential factor *A*, the activation energy *E*_*a*_ and the temperature exponent coefficient *n* are the parameters in this equation required to describe the kinetics.

(4) $$k=A\cdot T^{n}\cdot \;\exp\left(\frac{-E_{a}}{RT}\right)$$

Throughout this work when we refer to either thermodynamic or kinetic properties, these are the underlying properties of interest.

In this article, we review the state-of-the-art in machine learning for property prediction of molecules and reactions. The first part deals with the discussion of the methods currently incorporated in kinetic model generators for the calculation of thermochemical and kinetic properties. After that, machine learning approaches are discussed by their three main pillars: the data, the representation of the data, and the mathematical model. This is followed by an assessment of alternative training methods that improve the prediction performance. More specifically, we focus on methods that allow training on multiple datasets. Eventually, we elaborate on the accuracy that can currently be achieved with machine learning and the impact of these accuracies on detailed kinetic models. We end the review with the current limitations that are encountered, hampering the implementation of machine learning in detailed kinetic models.

## 2 Classical methods for thermodynamic and kinetic property calculation

As mentioned in the introduction, fitting the properties to experimental data is unfeasible for large kinetic models. Therefore, *in silico* techniques to calculate these properties are frequently used. The most fundamental way to calculate thermochemical properties is via quantum chemistry. In this approach, geometry optimization and energy calculations of molecules are performed via designated quantum chemistry packages like Gaussian (Frisch et al. 2016), TurboMole (Furche et al. 2014), or ORCA (Neese 2012). For the geometry optimization step, fast density functional theory (DFT) methods are usually accurate enough. Once the optimal geometry is obtained, the energy of the structure must be calculated. For these energy calculations, the earlier used DFT methods are usually not accurate enough. Therefore, more accurate quantum chemical methods such as coupled cluster methods or CBS-QB3 (Montgomery et al. 1999) are required. The use of these more advanced methods comes at the expense of a higher computational time. Furthermore, to achieve chemical accuracy i.e., deviations lower than 4.184 kJ/mol, corrections on the initial result might be required to compensate for inappropriate assumptions. A common example of such an assumption is the harmonic oscillation approximation. Here, the vibrational partition functions are calculated by assuming a harmonic potential at the vicinity of the minimum. This approach only requires a computationally-friendly calculation of the vibrational frequencies, but may lack accuracy. One popular way to go beyond this approximation is using the 1D-hindered rotor scheme (Pfaendtner et al. 2007). In this scheme, the potential energy surface for the rotation around a bond is calculated for each rotatable bond. This increases the accuracy of the property calculation but comes again at the expense of a larger computational time since a lot of additional DFT-calculations must be performed for the calculations of the potential energy surfaces.

Next to gas-phase properties, solvation properties can also be calculated quantum chemically (Cramer and Truhlar 1999; Klamt 2011; Tomasi et al. 2005). Many implicit solvation models are available in popular quantum chemical packages, which can model solvent effects without much increase in the computational time (Cramer and Truhlar 1999). Popular examples of implicit models are the polarizable continuum model (PCM) (Miertuš et al. 1981) and the solvation model based on density (SMD) (Marenich et al. 2009). These methods, however, often lack accuracy. Another option is to model the solvent explicitly. This often yields more accurate results, but, due to the larger system size, requires more computational time. A third option is using the conductor like screening model for real solvents (COSMO-RS) method (Eckert and Klamt 2002; Klamt 1995; Klamt and Eckert 2000). This semi-empirical method calculates solvent properties by matching the quantum chemically calculated COSMO surfaces of the solute and solvent. This approach shows satisfactory results, but its performance on certain molecule classes such as radicals remains unclear.

In addition, quantum chemical calculations are also valuable for property prediction of compounds in catalytic reactions. In heterogeneous catalysis, adsorption properties of reactants and products are required for the development of heterogeneous catalytic models. These properties can be determined ab initio but are challenging to predict as (I) the adsorption site is often ill-defined, (II) the obtained values generally have lower accuracy, (III) and the calculations are much more computationally intensive. Here, we will elaborate on the nature of these three challenges. Within heterogeneous catalysis, it is often up to debate what the exact nature of the adsorbed species is. In metal catalysts, the type of site such as bridge, terrace, or edge determines the stability of the adsorbed complex. Moreover, the catalyst structure in operando conditions can differ from what is experimentally determined at other conditions. Also in zeolite catalysis, the location of the acid site in the framework influences the adsorption properties. It is unfortunately not straightforward to determine the exact structure of the active site as the exact location of the Bronsted acid site is often unknown. Second, the complex nature of the adsorbed complex limits the accuracy of ab initio calculations. Adsorption properties can be calculated statically (i.e., via transition state theory) at a DFT level of theory. However, these approaches often fail to predict the adsorption entropy accurately, even though heuristics exist (De Moor et al. 2011a). For zeolite adsorption properties typical accuracies are in the order of ∼8 kJ/mol (Berger et al. 2023), while more accurate methods exist for metal sites (Sauer 2019). To overcome this shortcoming, molecular dynamics calculations, in which the geometry and energy of a species is tracked over time, can be performed which allow to achieve chemical accuracy. These increase the accuracy of the calculations, but also significantly increase the computational cost.

Kinetic properties can be obtained by following the same procedures described above for the reactants and transition state, as presented by transition state theory (Truhlar et al. 1996). Finding the correct transition state structure is significantly more challenging than finding the geometry of a stable species. This is because finding a transition state structure requires a good initial guess, which is hard to automate and therefore often requires human intervention. A bad initial guess could namely result in finding a too energetic saddle point or not converge to a saddle point at all. Overall, the quantum chemical procedure thus consists of many time-consuming steps. For reaction networks containing thousands of molecules and tens of thousands of reactions, these calculations are unfeasible, certainly if they require human interventions. Therefore, less accurate but faster and automated methods are often relied on to calculate thermochemical and kinetic properties.

The most popular computationally friendly approach to calculate molecular properties is group additivity, introduced by Benson et al. (1969). This method relies on the assumption that the thermodynamic properties of a molecule can be calculated by summing a certain contribution from every group in the molecule. The contribution that every group gives is usually obtained by regression towards ab initio or experimental values. The downside of this approach is that every group contribution is constructed based on the local neighborhood of that group, ignoring longer-range interactions. This problem has been partially mitigated by adding correction terms like non-nearest-neighbor interactions and ring strain corrections (Cohen 1996). Although these corrections improve the predictions, the accuracy might be insufficient for certain molecules, especially complex structures like polycyclic molecules. Also for the fast prediction of solvation properties, different methods have been proposed. Mintz and coworkers (Mintz et al. 2008; Mintz et al. 2009), for example, introduced a linear free energy relationship to predict the enthalpies of solvation. Group additive methods have also been applied to predict solvation properties (Khachatrian et al. 2017). The downside of these methods is that they only consider one solvent or one class of solvents. Consequently, the fitting procedure must be repeated for every new solvent or solvent class. Likewise, group additive approaches have also been developed for adsorbed species to calculate the adsorption energy (Gu et al. 2017; Salciccioli et al. 2010; Wittreich and Vlachos 2022). One major drawback of this approach is that new group additive values (GAVs) are required for every catalyst surface. A d-band model (Greeley et al. 2002; Hammer and Nørskov 1995) can be used to extrapolate toward other catalysts but has some limitations (Esterhuizen et al. 2020; Gajdoš et al. 2004; Vojvodic et al. 2014; Xin and Linic 2010; Xin et al. 2014). Furthermore, group additive models have also been developed for zeolite frameworks. Yu et al. (2023) developed a group additive method for the estimation of thermodynamic properties for a wide range of compounds relevant to methanol-to-olefins in a SAPO-34 catalyst. Besides group additivity, other linear relationships have been developed for the estimation of adsorption properties in heterogeneous catalysts. For example in zeolites, De Moor et al. (2011b) and Nguyen et al. (2011) found a linear relation between the adsorption energy and the number of carbon atoms for both linear paraffins and linear olefins, which have been shown to be also accurate for branched hydrocarbons (Denayer et al. 1998). In another approach, taken by RMG-cat, an automatic catalytic reaction network generator, adsorption properties are estimated based on the similarity of the queried compound and an existing library. This library comprises small hydrocarbons, nitrogenates, and oxygenates on metal sites (Goldsmith and West 2017).

There are many methods to predict kinetic properties in a fast manner. Evans and Polanyi introduced a famous linear free-energy relationship to predict the activation energy of a reaction based on the reaction energy (Evans and Polanyi 1936). This relationship, which has been used extensively in kinetic models (Froment 2013), has been extended to include more effects (Roberts and Steel 1994), and account for non-linear relationships (Blowers and Masel 2000). Similar to thermodynamic properties, kinetic properties can be calculated via group additive methods. A popular approach to employ group additivity in kinetics is to define a reference reaction with a corresponding value for a given reaction family. GAVs are calculated for all possible structural changes to the surrounding groups of the reactive center (with respect to the reference reaction) (Atkinson 1987; Saeys et al. 2004; Sabbe et al. 2008b). The target kinetic property then equals the sum of the value of the reference reaction and all group contributions of the made structural changes. Note that this is different from the group additivity scheme used for the calculation of thermodynamic properties. Here, for the calculation of kinetic properties, only groups in the surrounding of the reactive center (usually the atoms in the alpha-position) are considered, while for thermodynamic properties, all groups in the molecule are considered. This approach has been used to calculate activation energies, as well as pre-exponential factors of reactions (Paraskevas et al. 2015, 2016; Sabbe et al. 2008b, 2010; Van de Vijver et al. 2018). Another popular approach to predicting kinetics is rate rules. In this approach, a rule to calculate the rate of a certain type of reaction is constructed. A ‘type of reaction’ is here usually defined as all reactions with a certain substructure in and around the reactive center. The rate rule for such a reaction type can range from an Evans-Polanyi relationship to more complex rules (Johnson and Green 2024).

Overall, these fast methods to calculate thermodynamic and kinetic properties are significantly less accurate than the quantum chemical results on which they are based. Quantum calculations often do yield satisfactory results but are computationally too expensive, in particular for large kinetic models. Machine learning, on the other hand, is a promising technique to obtain fast predictions that are closer to quantum chemical accuracy than the traditional approximative approaches.

## 3 Machine learning for molecular property prediction

In this and the next chapter, machine learning methods to predict thermodynamic and kinetic properties will be discussed. Machine learning models transform the input (molecule or reaction) into the targeted output (thermodynamic or kinetic properties). During the training step, the model learns how to predict the output by regression toward training data. Once the model is trained, its performance can be assessed by evaluating the predictions of a test dataset. The quality and the amount of the data thus have a strong influence on the final performance of the model. Data is thus the first important pillar for the creation of machine learning models. This data (training or test) can usually not be fed to the machine learning model ‘as is’. First, it needs to be represented in a way that can be treated by a machine learning model. This introduces the second pillar: representation. Once the data (training or test) is converted to a suitable representation, it is fed into a machine learning model. The choice of the mathematical model is also an important step in generating machine learning models. Therefore, model choice is the third and last pillar on which machine learning models are built. In this and the following chapter, machine learning models for the prediction of molecular and reaction properties will be described via these three pillars.

### 3.1 Thermodynamic datasets

The first step in creating a machine learning application is collecting data. This is one of the most important elements determining the success of the machine learning model as low-quality or sparse data is detrimental to the final model performance. Different datasets exist that contain a high amount of molecules, such as GDB-17 (Ruddigkeit et al. 2012) or the PubChem database (Kim et al. 2019). These datasets, however, only contain molecules, and no thermodynamic properties linked to them. Therefore, these datasets are not suitable for the prediction of thermodynamic properties. For machine learning methods aimed at the prediction of thermodynamic properties, the data must link the input of the model with the targeted output. One of the most popular datasets containing gas-phase thermodynamic properties is QM9 (Ramakrishnan et al. 2014). This dataset was constructed by first taking a subset of the GDB-17 dataset. More specifically, only non-ionic molecules with a maximum of nine heavy atoms (all atoms excluding hydrogen) are considered. Furthermore, all molecules containing atoms other than carbon, hydrogen, oxygen, nitrogen, and fluorine are also excluded from the subset. Lastly, all charged molecules except zwitterionic species are removed from the dataset. This resulted in the QM9 dataset containing 133,885 molecules, for which thermodynamic properties have been calculated using the DFT method B3LYP/6-31G(2df,p). In this way, the dataset links the 3D geometry of molecules with the following important properties: the zero-point vibrational energy, the internal energy at 0 K, the internal energy at 298.15 K, the enthalpy at 298.15 K, the free energy at 298.15 K, and the heat capacity at 298.15 K. The accuracy of the calculations was tested by comparing the atomization enthalpies in the dataset with enthalpies calculated by the more accurate G4MP2, G4, and CBS-QB3 methods. For all of these methods, the mean absolute difference in the enthalpy of atomization was around 20 kJ/mol. Other properties such as the energy of the HOMO and LUMO are also present in this dataset but are less relevant for kinetic modeling purposes. Besides the 3D geometry of molecules, line-based identifiers such as SMILES and InChI are provided. More details about how molecules can be represented will be given in the next section. Although this dataset has been used widely, it has some serious shortcomings regarding kinetic modeling. First, the achieved accuracy of the calculations is very low. Furthermore, the dataset contains a significant amount of less occurring species, such as molecules containing three- or four-rings. Lastly, the dataset only contains closed-shell neutral species, which is not suitable for radical mechanisms. The latter problem has been mitigated by the work of St. John et al. (2020). They constructed a dataset containing 40,000 closed-shell and 200,000 radical species. The closed-shell molecules in this dataset were constructed by taking all neutral molecules from the PubChem database. Only molecules containing carbon, hydrogen, nitrogen, and oxygen, with a maximum of 10 heavy atoms were considered. In contrast to the QM9 dataset, zwitterionic species are not present in this dataset. Radicals were generated by breaking all single, non-ring bonds of the closed-shell molecules homolytically. Thermodynamic properties were calculated for both the closed- and open-shell molecules using the M06-2X/def2-TZVP DFT method. This M06-2X functional is considered to be more accurate than the B3LYP functional used for the QM9 dataset. With this method, important thermodynamic properties such as enthalpy and free energies were calculated. The molecules in this dataset are represented by their 3D structures, as well as their SMILES string, similar to the QM9 dataset. For completeness, we note ANI-1 as another large dataset containing 20 million data points (Smith et al. 2017). These data points correspond to different off-equilibrium conformations of 57,462 small molecules. The usefulness of these off-equilibrium conformations is rather limited for direct machine learning of thermodynamic properties. This dataset, however, can be used to train neural network potentials. These kinds of neural networks predict the structure of the potential energy surface, which can then be used to optimize molecules and predict their properties. This technique is however outside the scope of this review. More information about machine learning potentials can be found in the following reviews (Behler 2021; Kocer et al. 2022; Manzhos and Carrington 2021). A downside of the discussed datasets is that the properties therein are calculated via DFT methods. As already indicated, more advanced quantum chemical calculations might be required to obtain sufficiently accurate predictions of thermochemical properties. These more advanced techniques are computationally more demanding and might require human interventions, for example, to perform the 1D-hindered rotor scheme. These difficulties prevent the construction of large databases with more accurately predicted properties. However, smaller datasets, usually not for machine learning purposes, have been constructed. A major disadvantage of this data is that it is spread around the scientific literature. It is therefore unfortunately challenging to collect all thermodynamic data present in the literature. Nonetheless, Table 1 summarizes a selection of dataset sources and their specifications.

**Table 1:** Selection of thermodynamic databases found in the literature.

Molecule type	Number of data points	Molecule representation	Properties	Method	Source(s)
Oxygenates (including radicals)	450	SMILES	Δ_*f*_*H*^°^ Δ*S*^°^ *C*_*p*_	CBS-QB3 + 1D-HR + SOC + BAC	Paraskevas et al. (2013)
Hydrocarbons (including radicals)	233	SMILES	Δ_*f*_*H*^°^	CBS-QB3 + BAC	Sabbe et al. (2005)
Hydrocarbons (including radicals)	253	SMILES + 3D geometry	Δ*S*^°^ *C*_*p*_	B3LYP/ 6-311G(d,p) + 1D-HR	Sabbe et al. (2008a)
Carbenium ions	165	Name	Δ_*f*_*H*^°^ Δ*S*^°^ *C*_*p*_	CBS-QB3 + 1D-HR + SOC + BAC	Ureel et al. (2023a)
Alkanes, alkyl hydroperoxides (including radicals)	192	SMILES	Δ_*f*_*H*^°^ Δ*S*^°^ *C*_*p*_	STAR-1D or STAR-1D_DZ	(Ghosh et al. 2023b, a)
Oxygenated polycyclic aromatic hydrocarbons (including radicals)	92	Name + 3D geometry	Δ_*f*_*H*^°^ Δ*S*^°^ *C*_*p*_	G3 + 1D-HR	Wang et al. (2023)
Molecules relevant to atmospheric chemistry	323	3D geometry + Lewis structure	Δ_*f*_*H*^°^ Δ*S*^°^ *C*_*p*_	G3	Khan et al. (2009)
Silicon-hydrogen compounds	135	Lewis structure	Δ_*f*_*H*^°^ Δ*S*^°^ *C*_*p*_	G3	Wong et al. (2004)
H, C, O, N, and S containing species	371	InChI	Δ_*f*_*H*^°^	CBS-QB3 + 1D-HR + SOC + BAC	Pappijn et al. (2021)
Small combustion molecules	219	Name + Lewis structure	Δ_*f*_*H*^°^ Δ*S*^°^ *C*_*p*_	RQCISD(T)/cc-PV∞QZ + 1D-HR + SOC + BAC	Goldsmith et al. (2012)
Cyclic hydrocarbons and oxygenates (including radicals)	3,926	SMILES + InChI	Δ_*f*_*H*^°^ Δ*S*^°^ *C*_*p*_	CBS-QB3 + SOC + BAC	Dobbelaere et al. (2021a)
Radicals containing C, O, and H	2,210	SMILES	Δ_*f*_*H*^°^ Δ*S*^°^ *C*_*p*_	CBS-QB3 + AEC + BAC	Pang et al. (2024)
H, C, O containing species	1,340	SMILES + InChI	Δ_*f*_*H*^°^ Δ*S*^°^ *C*_*p*_	G3 + 1D HR	Yalamanchi et al. (2022)
Halocarbons (including radicals)	16,813	SMILES	Δ_*f*_*H*^°^ Δ*S*^°^ *C*_*p*_	G3 + 1D HR	Farina et al. (2021)

Only datasets containing enthalpy of formation, standard entropy, and heat capacities are considered. The following abbreviations have been used: 1D-HR, 1D-hindered rotor; SOC, spin-orbit corrections; BAC, bond additive correction; AEC, atom energy corrections.

One downside of these different data sources is shown in the ‘Method’ column of Table 1. Since there is not one gold standard method to perform quantum chemical calculations, these calculations are often performed at different levels of theory. The different (biased) errors of these methods introduce an additional challenge in the subsequent training of the machine learning model. Another shortcoming of this data is the gaps in the molecular space. Combining the datasets in Table 1 will namely miss important molecule classes, such as species containing both oxygen and a halogen atom or ionic species other than hydrocarbons. To identify these gaps and to gather data from various sources, different initiatives have been started to develop large databases from literature data. The RMG database, for example, combines data from 45 different libraries (Johnson et al. 2022). Another example containing enthalpies of formation is the Active Thermochemical Tables (ATcT) (Ruscic et al. 2004). A downside is that for these collected datasets, different calculation methods are used. Datasets containing experimentally measured values can overcome this problem. The NIST Computational Chemistry Comparison and Benchmark Database (CCCBDB) contains, next to computational values, experimental thermochemical properties of more than a thousand molecules. Similarly, the commercial DIPPR database contains around 2000 molecules with the corresponding experimentally measured enthalpy of formation and entropy (Bloxham et al. 2021; Thomson 1996). However, overall, a large dataset containing accurately predicted or measured thermochemical properties does not exist at this moment. This limited size of the accurate datasets is one of the reasons that makes traditional methods such as group additivity still the most popular choice to predict thermodynamic properties while making detailed kinetic models.

All previously presented datasets comprise thermochemical properties of gas-phase molecules. To include machine learning in liquid-phase kinetic models, databases containing the Gibbs free energy of solvation are essential. The Minnesota Solvation database contains 3037 solute-solvent pairs for which the free energy of solvation has been experimentally determined (Marenich et al. 2020). For both the solvent and solute the 3D coordinates, calculated at the M06-2X/MG3S level of theory, are included. Similarly, the CompSol database contains experimental free solvation energies at different temperatures and pressures for 14,102 solvent-solute pairs (Moine et al. 2017). Another literature dataset is FreeSolv, published by Mobley and Guthrie (2014). This dataset contains 643 small molecules for which the hydration free energy in water has been measured. Vermeire and Green (2021) combined the aforementioned datasets and an additional dataset developed by Grubbs et al. (2010) into one big database, comprising 10,145 solvent-solute pairs including 291 solvents and 1,368 different solutes with their experimental Gibbs free energy of solvation. Along with this experimental dataset, they have also developed a quantum chemically calculated dataset containing one million solvent-solute combinations. The Gibbs free energies of solvation in this dataset were calculated using the COSMO-RS methodology described in Section 2. Using this methodology, the calculation of the thermodynamic properties of *N* different species in *M* different solvents only requires *N* + *M* quantum chemical calculations.

This combinatorial advantage is not present for adsorption properties. Different databases exist for the properties and structure of catalytic materials such as metal catalysts, zeolites, and metal-organic-frameworks (MOF) (Jain et al. 2013; Kirklin et al. 2015). However, these databases do not include adsorption properties. The determination of the adsorption energies of *N* species on *M* different surfaces usually requires *N* × *M* quantum chemical calculations, making the construction of large databases time-consuming. Similar to gas-phase species, catalytic thermodynamic data is often spread around different publications (Andersen et al. 2019; Dickens et al. 2019; Esterhuizen et al. 2020; García-Muelas and López 2019; Schmidt and Thygesen 2018; Xu et al. 2021). However, also for surface species, databases that combine different data sources have been developed. One example is the pGrAdd software of the Vlachos group (Wittreich and Vlachos 2022). The package allows the calculation of thermodynamics based on group additivity. For these group additive schemes, data of surface species have been collected. This dataset contains standard enthalpies, entropies, and heat capacities, mainly of species on the Pt(111) surface. Another example is the Catalysis-Hub project (Winther et al. 2019). This project collected reaction energies, including adsorption energies, from more than 50 publications in one database. Similarly, a collection containing experimental datasets was constructed by Wellendorff et al. (2015). However, the limited size of this dataset makes it unusable for training large machine learning models. A bigger dataset was created in the Open Catalyst project, namely the OC20 dataset (Chanussot et al. 2021). This dataset was created by performing DFT relaxations on 1,281,040 catalyst-adsorbate combinations. In this publication three community challenges were also launched, each with their designated dataset. The first task is to predict the energy of and the forces on a (non-optimized) geometry. The second challenge is to predict the relaxed structure starting from the initial geometry. These two tasks are thus mainly relevant for the development of neural network potentials. This field of machine learning is, as mentioned before, out of the scope of this review. The third task, namely the prediction of the relaxed energy from the initial structure, is more relevant for this review. The dataset corresponding to this task links hundreds of thousands of initial geometry guesses to their relaxed energy and is therefore very relevant for the direct prediction of adsorption energies. Predicting the energy from an initial geometry guess could namely mean that the adsorption energy could be calculated in fractions of a second. In general, surface species data faces the same limitations as gas-phase data, but even stronger due to the increased computational complexity of determining adsorption properties. The data is often spread around literature and only encompasses a certain range of the molecular space. Furthermore, most databases are constructed by performing static quantum chemical calculations, instead of the more accurate molecular dynamics approach. The absolute accuracy of this data can therefore be questioned.

Overall, there clearly are limitations when looking at the data pillar for the prediction of thermodynamic properties. Large datasets already exist, but are usually calculated at a low level of theory. More accurate data is spread around the literature and contains important gaps i.e., for some molecule classes there is no accurate data available. Liquid-phase properties are less available than gas-phase data. Nonetheless, large datasets describing solute-solvent pairs and their free energy of solvation exist. This is not the case for adsorbed species on catalytic surfaces, for which there is little data describing their thermodynamic properties around literature and where quantum chemical calculations still lack accuracy to obtain chemically accurate properties at a reasonable computational cost. The collection of data is thus a major challenge in the creation of machine learning models predicting thermodynamic properties.

### 3.2 Molecular representation

Once the data is obtained, the molecules need to be computationally represented for the machine learning model. An ideal computational representation should answer to certain criteria which will be outlined here. A first desired property of a representation method is its uniqueness. This means that one molecule, using a representation method, can only be represented in one manner. If this is not the case, one molecule may be represented in two different ways, leading to two different property predictions by the machine learning model. This property may seem trivial, but in what follows, an example will be shown for which this is not the case. Secondly, the representation must be unambiguous. This means that a certain representation can only correspond to one molecule. If not, two molecules with the same representation will always get the same prediction from the machine learning model, which is clearly undesirable. A third important factor is that the representation must be easy to generate. The aim of the machine learning models discussed here is to obtain a fast prediction of the thermodynamic properties. If the representation step in this process takes a long time, the main advantage of machine learning is lost.

One of the most common ways to represent molecules is line-based string identifiers. These are identifiers in which a molecule is represented by a single string. The most used line-based identifier is the simplified molecular input line entry system (SMILES) string (SMILES – A simplified chemical language). This SMILES string describes a molecule unambiguously and is easy to generate. However, the SMILES string is not unique i.e. for one molecule many correct SMILES can be generated. This shortcoming can be remedied by using canonical SMILES, for which a mathematical algorithm re-orders the atoms and corresponding string, making it a unique representation. A challenge with this representation is that it is based on the bonds between the atoms. Deriving the correct bonds and bond orders may be challenging when only the 3D coordinates of the atoms are known. Another popular string-based method to identify molecules is the International Chemical Identifier (InChI) (Heller et al. 2015). This representation is unique, unambiguous, and easy to generate. A downside of InChI with respect to SMILES is that it is less human-readable. A third string-based representation of molecules that is worth mentioning is SELFIES (Krenn et al. 2019). The major advantage of this representation is that it is robust, meaning that when certain grammar rules are followed, every possible SELFIES string is related to a valid molecule. Therefore, this representation is promising to be used in generative machine learning models. However, due to its novelty, it has not been widely used in the general representation of molecular data, or as input for predictive machine learning models (Krenn et al. 2022). The aforementioned line-based identifiers only represent molecules in a 2D manner. They are in fact unambiguous using a 2D view but may be ambiguous when considering 3D conformations of molecules. Different conformers will namely be represented in the same way.

To properly represent a conformer of the molecule, 3D information must be incorporated into the representation. It is unfeasible to store all the 3D information i.e., the coordinates of the atoms, in a string. Therefore, a 3D molecule is usually represented via specific file formats such as xyz-files, mol-files, or sdf-files. While all these text-based representations (string or file) are readable for computers, they can usually not be used directly as input to a machine learning model. An exception to this is the recently emerging language models. These models can directly use the SMILES representation as input.

**Figure 3:**
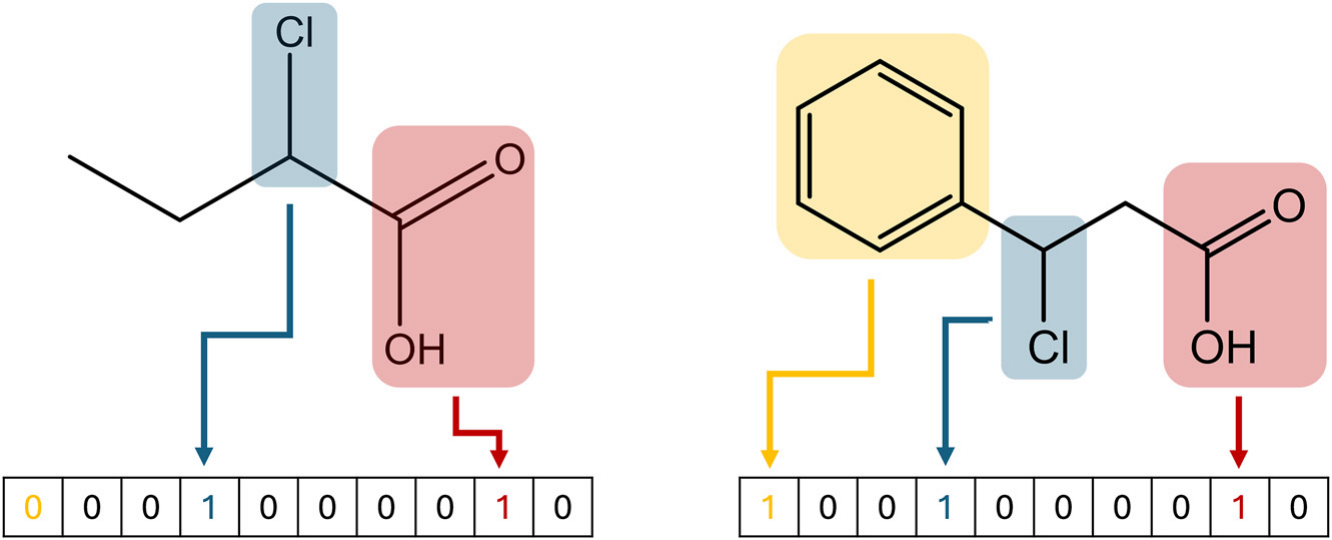
Generation of the MACCS key for two different molecules. Every element in the vector corresponds to a different substructure. If the substructure is present in the molecule, the corresponding value is set to 1. Else, the value is set at 0.

However, mostly, the aforementioned representations should first be converted to some sort of mathematical representation of the molecule. One of the most common representations for machine learning purposes is the numerical vector. Different features, such as molecular mass or number of atoms can be chosen as elements of this vector. It is important to consider the ambiguity when constructing vectors in this manner. Only selecting the molar mass and number of atoms would namely lead to many molecules having the same representation. Furthermore, the chosen feature must be easy to calculate. For example, using quantum chemical properties of the molecule would slow down the representation step significantly. Over time, many open-source and commercial packages to automatically generate such properties have been developed. Examples of such tools include Mordred (Moriwaki et al. 2018), ChemoPy (Cao et al. 2013b), Dragon (Mauri et al. 2006), and others (Yap 2011; Cao et al. 2013a). By using these tools, users can create vectors containing up to thousands of features in a reasonably short time period and without much manual intervention. In addition to these features, also structural features can be added. The structural features describe the presence or count of a substructure in the molecule. A popular way to include these substructures is the molecular access system (MACCS) key. This key encodes 166 substructures into a single representation vector, as shown in Figure 3.

This is an example of a well-established fingerprint that can be used for various purposes. These fingerprints are often included in cheminformatic packages such as RDKit (Landrum 2013), OpenBabel (O’Boyle et al. 2011), and CDK (Steinbeck et al. 2003). Other examples of such fingerprints are the RDKit fingerprint and the extended-connectivity fingerprint (ECFP) (Rogers and Hahn 2010). This ECFP fingerprint starts by assigning an initial representation to each atom. For a user-defined number of iterations, each atom representation is then updated based on the representations of the neighboring atoms. In this way, each atom has a final representation not only describing itself, but also its environment. These atom representations are then combined to obtain one molecular representation. The advantage of these built-in fingerprints is that they do not require expert knowledge. Furthermore, these fingerprint methods have the advantage of being unique and easy to generate. However, in some cases, for example, if two molecules contain the same groups or substructures, the representation might be ambiguous. Another downside is that these representations are not tailored to the targeted purpose. Furthermore, they do not include 3D information of the molecule.

**Figure 4:**
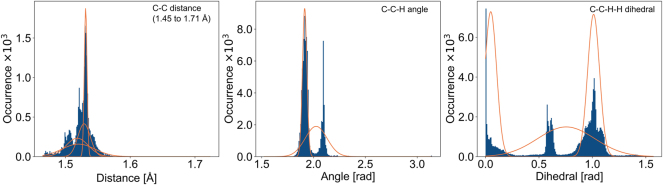
Histograms and fitted Gaussians of the C–C distance, the C–C–H angle, and the C–C–H–H dihedral angle.

One classical way of including 3D information is using Coulomb matrices (CM) (Montavon et al. 2012; Rupp et al. 2012). The diagonal elements of this matrix represent the atoms, and the off-diagonal elements contain the Coulomb repulsion between two nuclei. An advantage of using such a representation of the geometry is that it is invariant to external translations or rotations. Usually, a machine learning model requires a fixed-length vector as input. Therefore, this matrix must first be converted to a fixed-size matrix. This can be done by adding zeros (zero padding) until the desired matrix size is obtained. To transform this matrix into a vector, one can list all the elements of the matrix in vector form. More often, the eigenvalues of the matrix are calculated and put into a fixed-length vector (Montavon et al. 2012). Ordering the eigenvalues in descending order makes the representation invariant to atom numbering. Since the representation is now invariant to translation, rotation, and numbering, it is a unique representation of the molecule. Furthermore, the representation is easy to generate and unambiguous, even using a 3D view. Another method for adding geometrical information in the molecular representation is using histograms of distances, angles, and dihedrals (HDAD), first introduced by Faber et al. (2017) and further developed by Dobbelaere et al. (2021a). First, histograms are made of all distances, angles, and dihedral angles between atom types. Then, for each histogram, a number of Gaussians is fitted as shown for three histograms in Figure 4. After that, a vector containing the probability that a feature is found under each Gaussian is created for each geometric feature (distance, angle, dihedral). This vector has a length equal to the total number of Gaussians. These vectors are then added to obtain a representation vector of the molecule. A disadvantage of this approach is that the representation of a molecule is dependent on the dataset in which it is included. Again, this representation is invariant to translation, rotation, and atom numbering of the molecule, and is thus unique. Furthermore, it describes a molecule unambiguously in a 3D manner. Fitting the Gaussians over the histograms may be challenging, but once this is performed, the representation vector is also easy to determine. Besides the two geometrical representation methods presented here, many other approaches can be used (Faber et al. 2017; Hansen et al. 2015; Plehiers et al. 2021).

The earlier mentioned ECFP is a fingerprint that is based on the graph representation of the molecule. In this molecular graph, every node corresponds to an atom of the molecule, and every edge corresponds to a bond of the molecule. Once this graph is created, *a priori* defined operations are performed on the graph to obtain a numerical representation of the molecule. However, since the use of graph neural networks (GNNs) has recently become more popular, the transformation to a numerical vector is no longer needed. GNNs can namely take graphs as input to predict molecular properties. Therefore, next to string and vector representations, a graph is the third way to represent a molecule for machine learning purposes. For a molecular graph to be suited as input of a GNN, feature vectors must be assigned to the nodes (atoms) and/or edges (bonds). Common atom features to include in the vector are atomic number, number of bonded hydrogen atoms, number of non-hydrogen bonds, and implicit valence (Pathak et al. 2020; Rogers and Hahn 2010; Yang et al. 2019). Also more chemically inspired features such as electronegativity can be added. The most common choice of bond feature is the bond type i.e., single double, triple, and aromatic. This can be extended to more specific features such as whether the bond is conjugated or whether the bond is in a ring (Pathak et al. 2020; Yang et al. 2019). It is also possible to include 3D information of a molecule in its graph representation (Gasteiger et al. 2020). Gilmer et al. (2017), for example, included an encoding of the bond length in the bond feature vector when the geometry of the molecule was available. These graphs give a unique representation of the molecule when the atomic number is chosen as an atom feature and the bond order as a bond feature. It is also unambiguous in a 2D view. If 3D information is added, it can also describe different conformers unambiguously. Furthermore, using cheminformatic packages like RDKit, the molecular graph and its features are also easy to determine. For predicting properties, these graphs are treated by GNNs. By doing this, a latent vector representation of the molecule is created. However, since this vector representation is created by a machine learning model, this will be discussed in Section 3.3. For completeness we mention that the representation graph is sometimes constructed in a different manner. In these cases the nodes still correspond to the atoms in the molecule, but the edges are assigned differently. Namely, an edge can be added between atoms (nodes) that are closer to each other than a user-defined cutoff distance (Batzner et al. 2022; Gasteiger et al. 2020; Schütt et al. 2018, 2021). Setting this cutoff distance very high can even lead to a fully connected graph. In graphs created with a cutoff distance bond orders cannot be used as edge features. Therefore, the user must rely on geometrical features such as the interatomic distance.

**Figure 5:**
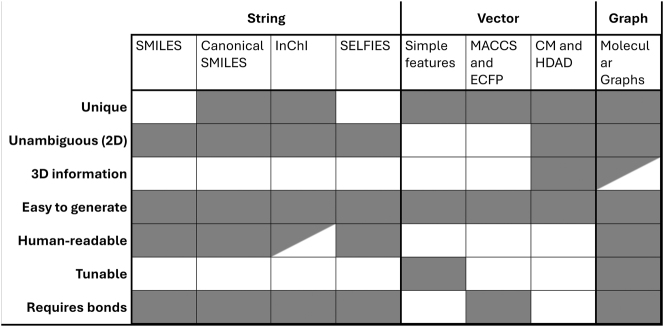
Overview of important properties of the discussed molecular representation methods. A cell is colored in gray if the representation follows the desired property. For the 3D information property the cell is half-filled if it is the user’s choice whether or not to include the 3D information. For the human-readable property, the cell is half-filled if it requires experience of the user to interpret the representation.

An overview of important properties of the discussed representation methods is shown in Figure 5. As mentioned before, unique means that one molecule corresponds to only one representation. Unambiguous means that one representation corresponds to one molecule (not considering conformers). The 3D information property shows if any conformational information is contained in the representation. In Figure 5, the box is half-shaded if it is the user’s choice whether to include it. A property is easy to generate if it can be created within fractions of a second. Note that if 3D information is used (like in CM, HDAD, and possibly graphs), the representation is only easy to generate if the geometry is already available. A representation is classified as human readable if it is simple to determine from the representation what the corresponding molecule is. For this category, half-shaded represents that it requires experience to deduce the initial molecule. Furthermore, a representation is tunable if the user can make choices in the representation, to tune it for the desired task. The last row shows if a representation requires bond knowledge to be generated. If this is required and only the 3D coordinates of the atom are given, the bonds in the molecules must be generated based on interatomic distances. These bonds can be assigned in different ways, influencing the uniqueness of the representation.

For the prediction of solvation properties in a single solvent, the molecular representation stated above can be used (Ferraz-Caetano et al. 2023; Goh et al. 2017; Hutchinson and Kobayashi 2019; Rong et al. 2020; Wu et al. 2018; Yang et al. 2019). However, for predicting solvation properties in a variety of solvents, a solvent representation must be created. Since solvents are molecules, they can be represented by a SMILES string. Again, this is usually not sufficient for machine learning purposes. To use it as input, the string must be translated into a numerical representation. One option is to embed both the solute and solvent in a feature vector (Chen et al. 2023; Liao et al. 2023a; Subramanian et al. 2020). Both the representation of the solute and solvent are then used as input for a machine learning model. In this case, care should be taken so that the representations are tailored to describe solvation effects. Solvation is namely dominated by intermolecular forces, while the gas-phase thermodynamic properties are determined by intramolecular interactions. An example of such a tailored representation is the COSMO*therm* feature vector. These features are well suited to describe solvation effects but require quantum chemical calculations. However, when the number of different solvents is low in comparison to the total number of data points, the few time-consuming quantum chemical calculations are justifiable. Another option is to represent both the solute and solvent with a graph (Chung et al. 2022; Pathak et al. 2020; Vermeire and Green 2021). Here, again, the atom and/or bond features are preferable specific to describe the solvation process. In principle, it is also possible to have a graph input for the solute and a vector input for the solvent. Machine learning models that can treat these inputs will be discussed in the next section.

For the prediction of adsorption energies, selecting features to construct a suitable representation of the catalyst is the most popular approach. Often, the d-band center and other density of state features are used together with some limited feature selection tools (Andersen et al. 2019; Fung et al. 2021; Goldsmith et al. 2018; Nayak et al. 2020; Toyao et al. 2018; Xu et al. 2021). The downside is the requirement of DFT calculations of the catalytic materials to obtain the molecular representation. This is thus only justifiable if the number of different catalysts is low in comparison to the total number of data points. To lower the computational cost, it is, however, possible to estimate these d-band features at a reduced computational cost (Noh et al. 2018). Even more computationally demanding than using d-band features is using DFT-calculated energies of certain species-catalyst combinations to calculate the adsorption energies of another species-catalyst pair (Andersen and Reuter 2021; García-Muelas and López 2019; Tran and Ulissi 2018). The generation of this representation is computationally less intensive than the quantum chemical determination of the adsorption properties for all adsorbate-catalyst pairs but is still too time-consuming for kinetic modeling applications. In addition to these computationally expensive representations, it is also possible to use easy-to-calculate features such as electronegativities and atomic radii (Andersen and Reuter 2021; Esterhuizen et al. 2020). Ideally, one does not need to compute ab initio properties as an input to obtain model predictions. Therefore, Xie and Grossman (2018) used the atom coordinates of the metal crystal as an input for their model to facilitate material property prediction. It should be noted that this is only an inexpensive representation if the geometry of the catalyst is already known. Also for the calculation of adsorption energies, graph representations have been used (Pablo-García et al. 2023). In this approach, the adsorbate and catalyst were encoded as one graph, and the node feature vector was a one-hot encoding of the corresponding element. Overall, by employing either the known geometry or other easy-to-determine features as model input, a much more user-friendly and faster prediction is achieved which is essential for the automatic generation of kinetic models.

In conclusion, there are three main types of molecular representation for machine learning purposes: string representation, vector representation, and graph representation. Also when looking at solvation or adsorption properties, molecules can be represented via a vector or graph representation. The advantage of the graph representation is that it contains a lot of information. It contains information about the atoms of the molecule, and with which bonds they are connected. Such a high amount of information is usually not contained in a vector representation. This is because all information must be compiled into a fixed-length vector. The least amount of chemical information is contained in a SMILES string. This might be counterintuitive since this SMILES is often the starting point of graph representations. However, for a machine learning model, the SMILES input is just a string without any additional meaning. The graph representation is thus the most complete representation of the molecule. However, in the next chapter, a major disadvantage of this graph representation will be touched upon.

### 3.3 Machine learning models for molecules

Following the view of Dobbelaere et al. (2021b), the third big pillar of machine learning, besides data and representation, is the machine learning model. The type of model that can be used depends on the type of representation that is chosen. If the molecule is represented by a numerical vector, a wide variety of machine learning models are suited for the task. Any model that transforms the input vector to the target output can be chosen. The simplest option is linear regression. However, due to their simplicity and linearity, these models are not within the scope of this machine learning review. Besides these linear models, more complex methods, such as support vector regression (SVR) (Dashtbozorgi et al. 2012; Yalamanchi et al. 2019, 2020), kernel ridge regression (KRR) (Faber et al. 2017; Noh et al. 2018; Rupp et al. 2012) or feedforward neural networks (FNN) (Dashtbozorgi et al. 2012; Dobbelaere et al. 2021a; Li et al. 2017; Yalamanchi et al. 2019) are often used for the prediction of thermochemical properties. When the molecule is represented by a mathematical graph, these classical methods are not suitable. In this case, GNNs are used. These are neural networks specifically dedicated to processing graph data. Many different GNNs have been developed to predict molecular properties (Wieder et al. 2020). Mostly, these models are based on iteratively updating the node representation based on its surroundings. However, other methods have been designed that update this representation based on the complete graph (Kearnes et al. 2016; Wu et al. 2018). Here, we will discuss message passing neural networks (MPNN), which is the most used architecture for predicting thermochemical properties (Ma et al. 2020; Wieder et al. 2020). As discussed in the representation section, the input to such models is a graph *G*. Each node in this molecular graph has an associated feature vector. This is a requirement if a node-centered MPNN, which is the most occurring type, is used. This feature vector will be denoted as *x*_*v*_, where *v* is the node of which this is the feature vector. Often, also the edges have an associated feature vector. The vector of the edge between node *v* and *w* will be denoted as *e*_*vw*_. In node-centered MPNNs, every node *v* has a hidden state $h_{v}^{t}$ at timestep *t*. This hidden state is updated every iteration. First, the hidden state must be initialized, as shown in equation (5).

(5) $$h_{v}^{0}=\text{init}\left(x_{v}\right)$$

This initialization function can be as simple as init(*x*_*v*_) = *x*_*v*_. However, then, the size of the hidden state $h_{v}^{0}$ is fixed to the same size as *x*_*v*_. This limits the number of hyperparameters that can be tuned by the user. For this reason, and to give the model flexibility in constructing these initial hidden states, learned matrices are usually used for this initialization (Ma et al. 2020; Hasebe 2021). In this context, ‘learned’ means that it can change during the training procedure. An example of such an initialization function is shown in equation (6), where *W*_init_ is a learned matrix, *b*_init_ a learned bias vector, and *σ* a nonlinear activation function. However, in general, this initialization function can be any function or even a neural network.

(6) $$\text{init}\left(x_{v}\right)=\sigma \left(W_{\text{init}}{\;x}_{v}+b_{\text{init}}\right)$$

After initialization, the iterative procedure of MPNNs starts. Every iteration consists of two stages: the message passing stage and the update stage. In the message passing stage, every node receives information from its neighboring nodes. The messages the node receives are then added to create the overall message $m_{v}^{t}$ received by node *v*, as presented in equation (7).

(7) $$m_{v}^{t+1}=\underset{w\in N\left(v\right)}{\sum }M_{t}\left(h_{v}^{t},e_{vw},h_{w}^{t}\right)$$

In this equation *N*(*v*) denotes the collection of all neighbors of node *v* and *M*_*t*_ the message function at iteration *t*. The function *M*_*t*_, which can be different for every iteration, is chosen by the user. The function can be as simple as $M_{t}\left(h_{v}^{t},e_{vw},h_{w}^{t}\right)=h_{w}^{t}$ (Duvenaud et al. 2015). However, mostly this message function contains learned matrices or is a complete neural network. Once every node has received an overall message, its hidden state must be updated based on this message, as shown in equation (8), in which *U*_*t*_ is the update function at iteration *t*.

(8) $$h_{v}^{t+1}=U_{t}\left(h_{v}^{t},m_{v}^{t+1},x_{v}\right)$$

Again, the complexity of this update function can range from simple arithmetic operations to a neural network. One special, but frequently used update function is the gated recurrent unit (GRU) (Feinberg et al. 2018; Liao et al. 2019; Withnall et al. 2020). This learned unit, originally designed for recurrent neural networks, has found its popularity in GNNs in recent years. After *T* iterations, every node has a learned representation describing its environment. This approach thus very much resembles the earlier mentioned ECFP, with the significant difference that here, the final atom representations are learned, whereas in the ECFP procedure, the atom representations are fixed. Finally, the hidden states of all nodes are converted to the targeted thermodynamic property, as is shown in equation (9).

(9) $$\hat{y}=\text{out}\left(\left\{h_{v}^{T}|v\in G\right\}\right)$$

**Figure 6:**
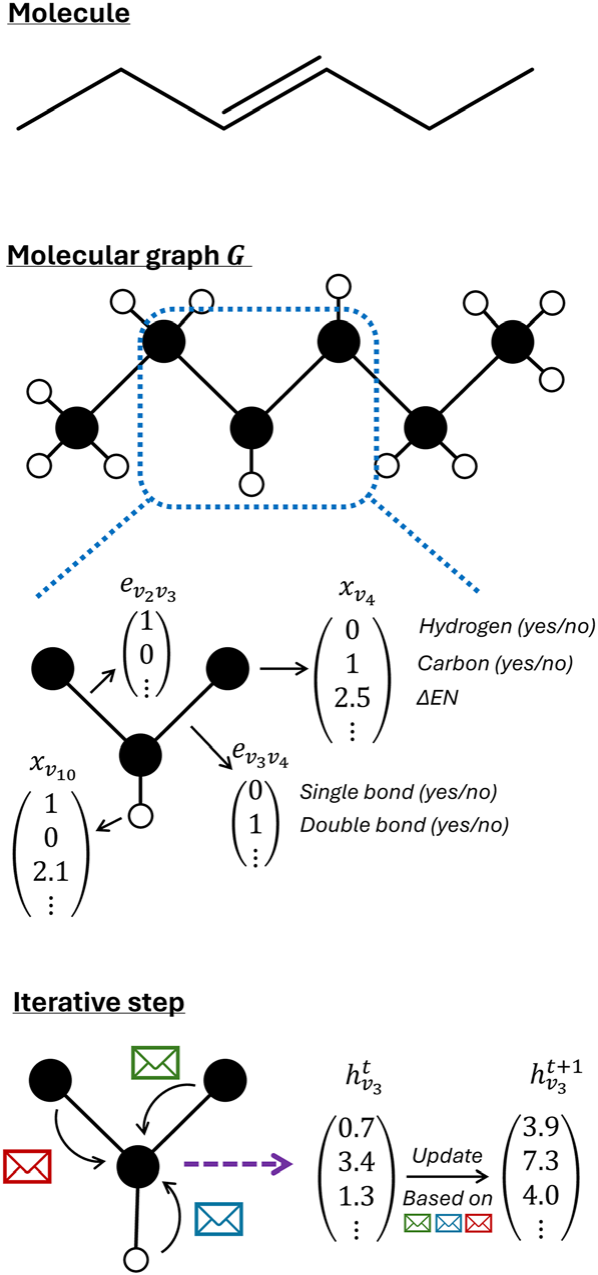
Representation of the construction of the molecular graph of 3-hexene, and a visualization of the iterative step in the MPNN.

In this equation, the output function out can be any function, learned or fixed, that transforms the node representations to the prediction(s). Mostly, it is a learned function in which the first step is adding all the hidden representations, $\underset{v\in G}{\sum }h_{v}^{T}$ , to obtain a single vector. It should be noted that this summed vector serves as a latent vector representation of the molecule. This latent representation is then fed to an FNN in the second step to obtain the prediction(s). Before feeding this latent representation to the FNN, it can be extended with additional features. These can, for example, be the temperature at which the target thermodynamic property is calculated/measured. Another option is to add features describing the solvent or catalyst (Heid and Green 2022). In this way, a graph representation of the molecule can be combined with a vector representation of the solvent or catalyst. Besides this popular approach, other output functions have also been proposed (Duvenaud et al. 2015; Schütt et al. 2017). The discussed method of converting a molecule in a graph and the iterative procedure that follows it is shown in Figure 6.

More details about MPNNs and GNNs can be found in the work of Gilmer et al. (2017) and the review of Wieder et al. (2020). One major advantage of using a graph representation in combination with a GNN is that the model can optimize the latent vector representation of the molecule for the given task by tuning the model parameters. Depending on the task and even the training data, the molecule will thus be represented by a different vector. Another advantage is that, since this vector representation is learned, expert knowledge is not really required to construct a machine learning model. A drawback of using GNNs is that they contain a high number of parameters and are as a consequence very data-intensive.

The last type of molecular representation that could be used as input for a machine learning model is the SMILES string itself. In these machine learning models, the SMILES strings are converted to a latent representation. Historically, recurrent neural networks (RNNs) were used to perform this task (Gómez-Bombarelli et al. 2018; Xu et al. 2017). Over recent years, great advances have been achieved in the field of natural language processing (NLP) (Devlin et al. 2018; Vaswani et al. 2017). The main novelty of these works is that language can be treated only using attention mechanisms, removing the need for RNNs. More details about this new technique can be found in the original publications (Devlin et al. 2018; Vaswani et al. 2017). The first part of such language models is usually a Bidirectional Encoder Representations from Transformers (BERT) (Chithrananda et al. 2020; Wang et al. 2019; Wu et al. 2022; Zhang et al. 2021). This encoder transforms a string, here the SMILES of the molecule, to a mathematical vector representation. After this BERT encoder, an FNN is usually used to predict the target property from the vector representation (Wang et al. 2019; Zhang et al. 2021). It is possible to train this combination of the BERT encoder and FNN via the conventional method. However, these two parts can also be trained separately. Alternatively, a transfer learning approach is frequently used to improve the prediction accuracy. This separate training and transfer learning approach will be discussed in a following section.

The models used for the prediction of solvation properties are similar to the ones used for the prediction of gas-phase properties. For the prediction of solvation energies in the same solvent for all data points, the solvent does not need to be represented, and the solute can be embedded similarly to gas-phase molecules. Therefore, the same model architectures can also be used. When the solvent differs along the dataset, the solvent must also be represented. When the solvent and solute are each represented by a feature vector, the vectors can be concatenated to obtain one vector describing the solute-solvent pair. This vector can then be used to calculate the target property using any of the aforementioned models that transform a vector into a numerical output (Chen et al. 2023; Liao et al. 2023a). If the solute and solvent are represented by a graph, GNNs can be used to treat them. After the iteration step, the latent representation of both are concatenated and then fed into an FNN (Chung et al. 2022; Vermeire and Green 2021). Also other techniques to predict solvation energies exist. For example, some works calculate interactions between atoms of the solute and solvent, mimicking the physical solvation process (Lim and Jung 2021; Pathak et al. 2020). Based on these interactions, the solvation energies are then calculated. As mentioned before, it is also possible to have a graph input for the solute and a vector input for the solvent. The solvent representation is then appended to the latent solute representation in the GNN.

The prediction of adsorption energies can be done in an analogous way to the prediction of solvation energies. In principle, the catalyst feature vector can be appended to the adsorbate descriptor. This larger vector can then again be used as input to a classical machine learning model (SVR, KRR, FNN) (Nayak et al. 2020; Toyao et al. 2018). However, most models for predicting adsorption energy are for a single adsorbate. Using the catalyst descriptor solely is thus sufficient in this case. Fung et al. (2021) created a machine learning model that could handle varying catalysts and adsorbates. After some steps latent feature vectors were obtained which were then concatenated. This vector was then fed into a feedforward neural network to obtain the adsorption energy prediction. Pablo-García et al. (2023) also predicted the adsorption energies of various adsorbate-catalyst combinations. The complete adsorbate-catalyst pair was embedded in a single graph. Therefore, a single GNN could be used to predict the adsorption energy. Besides this work, others have also used GNN to predict adsorption energies or related properties using a similar approach (Ghanekar et al. 2022; Li et al. 2023). While the number of machine learning models for direct thermochemical property prediction for heterogeneous catalysts remains limited, many efforts have been made in the area of neural network potentials for catalytic processes. Especially to predict the OC20 datasets, many machine learning potentials have been developed (Gasteiger et al. 2021; Liao et al. 2023b; Zitnick et al. 2022). Furthermore, important steps are being taken in model development for the prediction of crystal properties (Park and Wolverton 2020; Xie and Grossman 2018). Xie and Grossman (2018) developed a crystal graph convolutional neural network specifically for material property prediction. Park and Wolverton (2020) improved upon this model by incorporating information on Voronoi tessellated crystal structures. These types of models allow a complete and unique representation of the materials which is an important prerequisite for the prediction of adsorption energies in heterogeneous catalysts.

When selecting a model, an important consideration is its data requirements. Some models necessitate less data for training compared to others. For instance, training a large neural network (i.e., one with a high number of parameters) with a small dataset often leads to overfitting. Conversely, employing the same dataset to train a smaller neural network or simpler models like SVR or KRR tends to mitigate overfitting. This phenomenon can pose challenges when using a graph representation since it must always be paired with GNNs, which typically have numerous parameters. Thus, while a graph offers the most complete representation, it may not be optimal when data availability is limited. Using a string representation presents a similar issue. The BERT encoder, for example, comprises numerous parameters that require fitting, potentially resulting in overfitting. Nonetheless, as previously mentioned, alternative training methods can be employed to address this issue, which will be discussed in Section 5.

## 4 Machine learning for reaction property prediction

### 4.1 Kinetic datasets

Machine learning of reaction properties is less common in literature than the prediction of molecular properties. One of the main reasons for this gap is the availability of data. Reaction datasets are not as well established as molecular datasets. The first reason for this is the computational cost of constructing one. Constructing reaction databases usually requires searching, optimizing, and calculating the energy of transition states. This search and optimization procedure on transition states is computationally more demanding than it is on stable species. This higher computational cost leads to smaller datasets. Furthermore, the theoretical reaction space is larger than the molecular space (Stocker et al. 2020). Building a general machine learning model to predict properties for a wide range of inputs, would thus require more data when treating reactions, in comparison with having molecules as input. The lack of sufficient qualitative data is thus a major bottleneck for machine learning of kinetic properties. Nonetheless, efforts have been made to construct reliable datasets. One example is the datasets designed by the Green group (Grambow et al. 2020b; Spiekermann et al. 2022a). First, molecules involving hydrogen, carbon, nitrogen, and oxygen, with six or seven heavy atoms were selected from the GMB-7, which is a subset of the GDB-17 database. Starting from the optimized geometry of these molecules, transition states were sought via a single-ended method at the B97-D3/def2-mSVP level of theory. In single-ended methods, transition states are searched for starting from the reactant(s), while not having any knowledge about the products (Zimmerman 2015). After checking the validity of the transition state, the reaction energy and the activation energy were calculated at the B97-D3/def2-mSVP level of theory. This resulted in a dataset of approximately 16,000 reactions. To increase the accuracy, the geometry optimizations and energy calculations were reperformed at the ωB97-D3/def2-mSVP and the CCSD(T)-F12a/cc-pVDZ-F12 levels of theory for a dataset of approximately 12,000 reactions. Remarkable about this dataset are the types of reactions. Since the reactions are generated via an automatic single-ended method, a high variety of reaction types is obtained. This can both be an advantage and a disadvantage, depending on the aim of the machine learning model. A downside of these reaction datasets is that they only contain unimolecular reactions. Although the reactions can be reversed to obtain bimolecular reactions, the dataset still describes a limited range of the chemical reaction space. Either the reactants or products would consist of only one species. This limitation is not present in the work of Zhao et al. (2023b). They calculated the kinetics for almost 177,000 reactions. First neutral closed-shell molecules were selected from the PubChem database. The selected molecules consisted of C, H, O, and N, and contained no more than 10 heavy atoms. On these initial reactants, reactions are enumerated where two bonds are broken, and two bonds are formed. Both the reactant and the product are then used as input for a double-ended transition state search, at the GFN2-xTB level of theory. In such a double-ended search, a transition state is sought based on both the reactant(s) and product(s). Often, this search resulted in transition states relating to unexpected reactants and/or products. These unintended reactions were retained to increase the diversity of the dataset. Because of this, also bimolecular reactions and reactions breaking or forming less or more bonds are included in this dataset. For these reactions, the energetics were calculated at the B3LYP-D3/TZVP level of theory. In contrast to the previous dataset, not only reaction and activation energies were calculated, but also Gibbs free reaction and activation energies. A downside of the aforementioned reaction datasets is that the kinetic properties are calculated at a level of theory with a relatively low accuracy i.e., all but one are calculated using a DFT method. Furthermore, these datasets are constructed by automatically generating possible reactions. The first ones were constructed by searching for a transition state on the potential energy surface. The last database was constructed by breaking and forming bonds in the molecular graph. Both approaches may lead to reactions that are irrelevant or even unrealistic to occur in reality. The kinetics of a more relevant class of reactions was calculated by von Rudorff et al. (2020). They calculated the activation energy for thousands of E2 and S_N_2 reactions. These reactions are relevant in synthetic chemistry, but less prevalent in high-temperature reaction networks. Data considering high-temperature reactions is, similar to molecular data, usually spread around literature. Table 2 shows a selection of high-temperature reaction data calculated at a high level of theory that is available in the literature.

**Table 2:** Selection of high-temperature kinetic databases calculated at a high level of theory found in the literature.

Reaction type	Number of data points	Reaction representation	Properties	Method	Source(s)
Hydrogen transfer between oxygenates	118	Drawing + TS geometry	Arrhenius	CBS-QB3 + 1D HR	Paraskevas et al. (2015)
Radical addition and *β*-scission of oxygenates	66	Drawing + geometries	Arrhenius	CBS-QB3 + 1D HR	Paraskevas et al. (2016)
Intramolecular hydrogen abstraction	448	Drawing + TS geometry	Arrhenius	CBS-QB3 + 1D HR	Van de Vijver et al. (2018)
Carbon-centered radical additions and *β*-scissions	51	Drawing + TS geometry	Arrhenius	CBS-QB3 + 1D HR	Sabbe et al. (2008c)
Nitroethane flame reactions	729	Chemkin file format	Arrhenius	QCISD(T)/CBS	Zhang et al. (2013)
Tetrafluroropropene combustion reactions	1,530	Chemkin file format	Arrhenius	CBS-QB3	Needham and Westmoreland (2017)

Arrhenius properties include the pre-exponential factor *A*, activation energy *E*_*a*_, and may include the temperature coefficient *n*.

This table shows two types of data sources. The first four sources contain data to construct kinetic GAVs, whereas the last two sources contain data to construct detailed kinetic models. The advantage of the data generated for GAV purposes is that it only contains data of a strictly defined reaction class. This facilitates the generation of a machine learning model aimed at the prediction of the kinetics of that reaction class solely. The downside of these sources is their limited number of data points. On the contrary, when calculating kinetics for kinetic models, more data points are generated. However, these reactions span a wide range of reaction classes, which makes the creation of reaction class-specific machine learning models infeasible. Similar to molecular datasets, the RMG database has collected data from diverse sources in one database. Again, the downside of such a collected database is that the included properties may have a different calculation method and accuracy. The same issue is present in the NIST Chemical Kinetics Database (Mallard et al. 1992). However, the advantage of this database is that it, besides computed kinetic properties, also contains experimentally measured properties, which are generally more accurate.

Datasets concerning liquid-phase reactions are scarcer. One challenge in generating machine learning suitable databases is the size of the liquid-phase reaction space. Where the gas-phase reaction space was already considerably larger than the molecular space, adding solvents adds another dimension. Therefore, a high amount of data is required to generate a machine learning model that can predict kinetic properties throughout the complete liquid-phase reaction space. Constructing a machine learning model for only a part of the space requires less data, but will only cover a very small application range. An advantage is that in principle, generating datasets for liquid-phase reactions is not much more computationally demanding than gas-phase calculations, provided that the solvent effects are included in a simple manner e.g., implicit solvent model or COSMO-RS. Such an approach was taken by Stuyver et al. (2023). They calculated the free reaction energy and the free activation energy of around 5000 cycloaddition reactions in water. The energies were calculated at the B3LYP-D3(BJ)/def2-TZVP level of theory. The solvent effects were included in this calculation using the implicit SMD model. This dataset only covers a small fraction of the chemical space, since it only covers one reaction class and one solvent. Nonetheless, this is a good example of the type of data that is required to train machine learning models. A dataset that covers a larger portion of the reaction space was presented by Jorner et al. (2021). They collected around 500 rate constants of nucleophilic aromatic substitution reactions. The dataset contains reactions in different solvents and thus covers a significant part of the chemical reaction space. Furthermore, all rates are determined experimentally and are thus more reliable than computational values. In this work, the rates were also calculated quantum chemically. This resulted in a mean absolute difference of 12.26 kJ/mol on the free activation energy in comparison with the experimental values. This shows that statically calculated liquid-phase rates may not be accurate enough to construct quantitative detailed kinetic models as these values are not chemically accurate (below 4.184 kJ/mol). Nevertheless, combining these calculations with a machine learning model to predict the experimental rates lowered the error to 3.64 kJ/mol, showing the usefulness of these less accurate calculations. Other relevant liquid-phase reaction data was published by Chung and Green (2024). In this work, instead of performing the complete workflow of calculating of in-solvent reaction rates, only the solvent correction on the gas-phase reaction rate was calculated using the COSMO-RS theory. This resulted in a dataset of almost 8,000,000 datapoints, based on around 26,000 gas-phase reactions published by Grambow et al. (2020b), and a dataset of approximately 500,000 datapoints based on 1870 gas-phase reactions published by Harms et al. (2020). These large datasets show the strength of the COSMO-RS theory in the generation of large in-solvent reactions. Namely, after a quantum chemical calculation on the reaction species (reactants, products, and transition states) and the solvents, the solvent correction of many different reactions in many different solvents can be calculated relatively quickly. Databases of catalytic reactions face the same challenges as liquid-phase reactions. The catalytic material adds another degree of freedom to the already large chemical reaction space. Therefore, it is difficult to construct a dataset covering the complete, or a significant fraction of this catalytic reaction space. An additional challenge compared to liquid-phase reactions is the computational cost of generating datasets. Quantum mechanical catalytic calculations take, even when a static approach is taken, more computational time than liquid-phase calculations, due to the high number of atoms/electrons. Furthermore, for catalytic reactions, it is hard to exactly know the location of the transition state, which is less of an issue for gas-phase reactions. These limitations make the construction of large datasets containing computed catalytic reaction rates unfeasible. One place where catalytic reaction kinetics are available is the earlier mentioned Catalysis-Hub. This database contains, besides adsorption energies, reaction and activation energies of surface reactions.

In terms of data, the same problems are thus faced when predicting reaction properties as when predicting molecular properties. The scarcity of high-fidelity data is also a major problem when predicting kinetics, even more outspoken than was the case for molecular properties. This is mainly due to the higher computational cost of constructing such large datasets. Furthermore, since the reaction space is larger than the molecular space, more data is required to give a complete description of the space. Relatively large datasets still exist, but they contain properties calculated at a low level of theory. Similar to molecules, more accurate datasets are spread around the literature. For liquid-phase or catalytic reactions, only task-specific datasets exist.

### 4.2 Reaction representation

**Figure 7:**
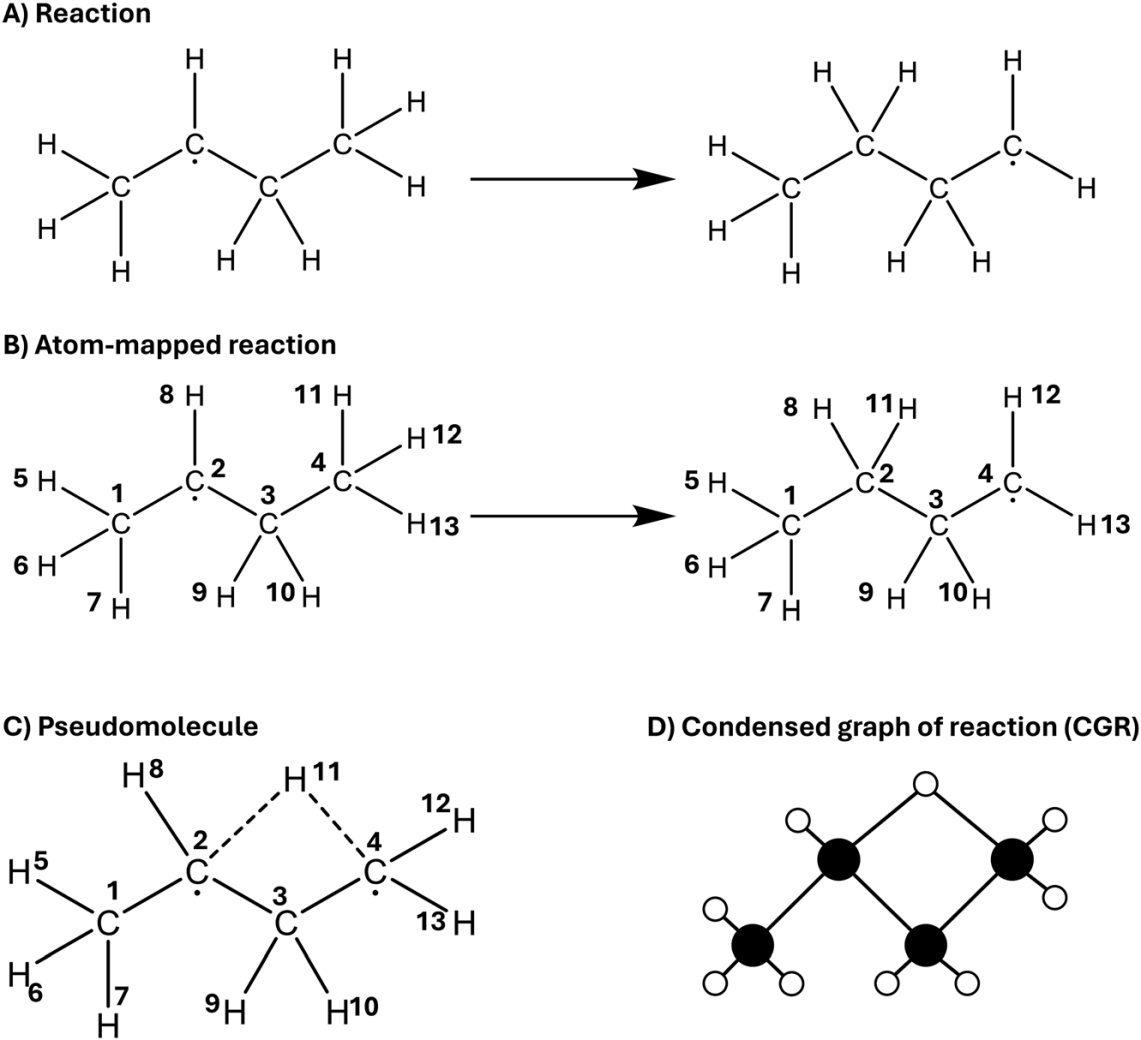
An example showing the need for atom mapping to represent a reaction unambiguously and the generation of the CGR. (A) The reaction of 2-butyl to 1-butyl, which can proceed via a 1,2-H shift and a 1,3-H shift. (B) The atom-mapped reaction, making clear that it is a 1,3-H shift reaction. (C) The pseudomolecule representing the reaction, in which the dynamic bonds are represented with dashed lines. (D) the CGR of the reaction (without showing the feature vectors associated with the nodes and edges).

A key step in creating machine learning models to predict kinetics is the representation of the chemical reactions. Representing reactions is challenging since there are some significant differences between molecules and reactions. A molecule is something static, whereas reactions are a dynamic process in which molecules are converted into each other. Ideally, a reaction would be represented by all atomic configurations along the reaction path. However, it is hard and memory-demanding to store reactions that way. Therefore, reactions are often represented by only their initial state (reactants) and end state (products), as shown in Figure 7A. This figure also shows a problem with this type of representation. Based on the representation, the reaction could be a 1,2-H shift or a 1,3-H shift. This reaction representation is therefore ambiguous. A machine learning model would predict the kinetic properties (e.g. activation energy) of the 1,2-H shift and the 1,3-H shift with these reactants and products as equal, which is not correct. A popular way to make the reaction representation unambiguous is to include atom mapping as shown in Figure 7B. In this approach, every atom in the reactants is linked with the corresponding atom in the products. With atom mapping, it is now clear that the reaction shown in the figure belongs to the 1,3-H shift reaction class. This representation, however, is merely a human-readable drawing of the reaction. For computational purposes, the reaction must be converted to a computer-readable format. The simplest way to achieve this is again using a line-based identifier like reaction SMILES (Reaction SMILES and SMIRKS) or Reaction InChI (RInChI) (Grethe et al. 2018). The reaction SMILES is constructed by separating the reactants’ and the products’ SMILES by a ‘>>’ sign. A major advantage of this representation is that, since every atom is represented separately in a SMILES string, atom mapping can be contained in the reaction SMILES string. Less frequently used is the RInChI representation (Grethe et al. 2018). The downsides of this representation are that it is less human-readable and cannot incorporate atom mapping. These line-based representations are usually not the input to a machine learning model, unless when working with an NLP model.

As was the case for molecules, reactions are frequently represented by a mathematical vector. Often, these reaction vectors are created from the molecular feature vectors of the reactants and products. One of the most common ways to do this is through difference fingerprints, introduced by Schneider et al. (2015). In this approach, the representation vectors of all reactants are added to obtain one representation vector of all reactants. The same procedure is performed for the products to obtain a vector describing all products. Finally, the reactants vector is subtracted from the products vector to obtain a reaction representation vector. This type of reaction representation, or others based on the difference between reactants and products, has been employed in many works (Ghiandoni et al. 2019; Patel et al. 2009; Probst et al. 2022). This popular method has several advantages. First of all, it is a flexible method. Any molecular feature vector can be used to generate the reaction vector. Secondly, after taking the difference between the reactants and products, only what changes during the reaction remains. The vector thus gives an intuitive representation of the reaction. Thirdly, this reaction representation does not require atom mapping. On the one hand, this results in a limited representation of the reactions, as was discussed before. On the other hand, not requiring atom mapping allows the use of reaction datasets for which mapping is not available.

Reactions can also be represented using mathematical graphs. A common way is using the molecular graphs for all reactants and products (Grambow et al. 2020a; Kwon et al. 2022; Wen et al. 2021). These graphs are then all used as input for a machine learning model. Since multiple graphs are used as input, customized machine learning models must be used. It is also possible to convert the reaction into a single graph, so that simple GNNs, as described in the molecular property prediction section, can be used. This single graph representation of the reaction is known as the condensed graph of reaction (CGR), based on the imaginary transition structure introduced in the 1980s (Fujita 1986). The CGR is constructed by converting every atom of the reactants or products into a node of the graph. Then edges are added between atoms that are bonded in either the reactants or the products. In this way, the reaction is represented as a type of pseudomolecule, as shown in Figure 7C (Hoonakker et al. 2011a). In this figure, the dashed lines represent dynamic bonds, which are bonds that change during the reaction (Hoonakker et al. 2011b). Just as for a molecular graph, feature vectors must be allocated to each node and edge. These vectors are created by combining features of the atom/bond in the reactants with its features in the products (Heid and Green 2022). In this way, changes during the reaction are incorporated into a single graph and classical GNN can be used. Note that for this graph representation, atom mapping is required to match the atoms in the reactants with the atoms in the products (Madzhidov et al. 2015).

Reactions can thus be represented by a string, a vector, or one or more graphs. For the prediction of liquid-phase or catalytic reaction kinetics, this representation must be combined with a representation of the solvent and catalyst, respectively. In the reaction SMILES string representation these reagents are represented in the following way: “*reactants > solvent.catalyst > products*”. For vector representations of the reaction, the solvent and/or catalyst can also be represented by a vector. The same solvent and catalyst representation methods for solvent and catalyst representation as applied in molecular property prediction can be used. Also when the reaction is represented by a graph, a vector representation of the solvent or the catalyst can be used. The input to the machine learning model is then one or more graphs and a vector. In the molecular property prediction section, it was already discussed how these can be combined in a machine learning model.

Similar to molecules, reactions are mainly represented by a string, vector, or one or more graphs. The representation of reactions is very similar to the representation of molecules because the reaction representations are often obtained by constructing the molecular representation of the reactants and products. The properties of the reaction representation (uniqueness, unambiguity, ease to generate) are usually linked with the corresponding properties of the used molecular representation method. However, special care should be taken to ensure unambiguity. To ensure the unambiguity of the reaction representation, it is important that atom mapping is included in the representation in any way. This can be done explicitly, for example in reaction SMILES, or implicitly, like in the CGR representation of a reaction.

### 4.3 Machine learning models for reactions

**Figure 8:**
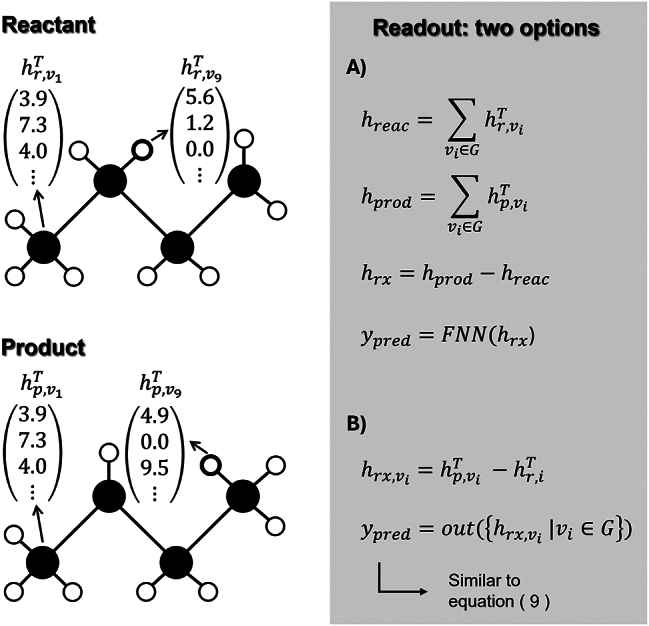
Visualization of the readout step of an MPNN when having multiple graph inputs by (A) creating latent reactants and products representations and combining them to make a latent reaction representation which is then fed into an FNN, or (B) creating an ‘atom reaction representation’ for every atom in the reactants/products. These are then fed into an output function similar to equation (9).

The preceding section highlighted that reactions are depicted using data structures previously introduced in the molecular representation section, allowing for the utilization of the same machine learning models. When a reaction is represented by a vector, conventional machine learning models like SVR, KRR, or FNNs are applicable. Similarly, when combined with a vector representation of the solvent or catalyst, these models remain suitable. Likewise, if the reaction is represented by a single graph, the aforementioned GNNs can be employed. When the input also contains a vector representation of the solvent or catalyst, this vector can be appended to the latent vector representation of the reaction. When the input consists of multiple graphs, a more specific architecture must be designed. Usually, all graphs are fed into a GNN to obtain a latent representation of every node(atom) and/or edge(bond) in the graphs. Then, there are two main methods to combine these representations to obtain a prediction of the target kinetic property. The first one is to first create the latent molecular representation of the reactants and products. These are then combined into one vector, e.g., by concatenating or subtracting them, which can then be fed into an FNN (Kwon et al. 2022; Wen et al. 2022), as shown in Figure 8A. When subtracting the latent molecular vectors, an identical approach to the earlier described representation method of Schneider and coworkers is taken, now incorporated in a machine learning model. The second option is first combining the node representations of the reactants with the corresponding node representations of the products (Grambow et al. 2020a; Wen et al. 2021). Then the same output functions as used in classical MPNNs can be used to convert these combined atom representations to the target property, as shown in Figure 8B. Note that to combine the representation of the corresponding atoms, atom mapping is required. For these two methods, again, possible vector representations of the solvent or catalyst can be added to the latent reaction representation. Another popular feature to add to this latent representation, when predicting the activation energy, is the reaction energy. The Evans–Polanyi relationship showed that there is a correlation between the reaction and activation energy. Adding this reaction energy can thus help the model in obtaining an accurate prediction of the activation energy. Also here, the reaction SMILES representation can be directly used as input of a machine learning model, using the BERT encoder. This BERT model, combined with an FNN has been used to predict yields of organic reactions by Schwaller et al. (2021b). Although the model is used for yield prediction, it can also be utilized to predict kinetics, as was also stated in the conclusion of this work. Also for reactions, the BERT encoder and the FNN can be trained separately or via a transfer learning approach. These approaches will be discussed in the following section.

## 5 Data-efficient machine learning

A challenge that remains is that classical machine learning approaches may lead to unsatisfactory accuracies, often because of a lack of good-quality data. Therefore, techniques have been developed to improve the performance of these models despite a low amount of adequate data. Here, we will discuss several approaches to achieve these improved accuracies.

**Figure 9:**
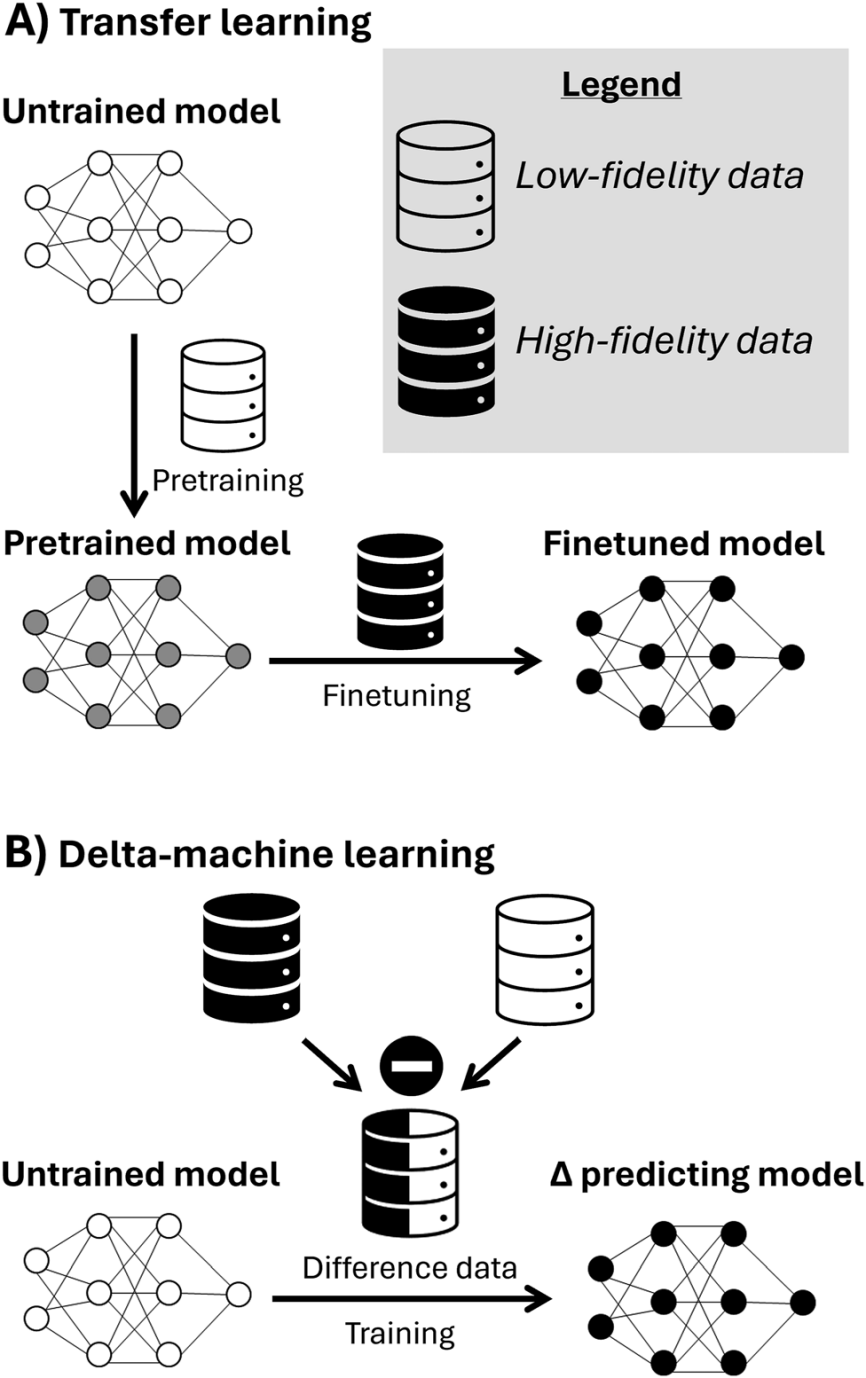
Two data-efficient machine learning techniques. (A) The transfer learning method. The black dataset represents the high-fidelity dataset, the white dataset is the low-fidelity dataset. (B) The delta-machine learning approach. The representation of the datasets is identical to (A). The black-and-white dataset represents the difference between the high-fidelity and low-fidelity data.

A first popular technique is transfer learning. The term transfer learning can be interpreted in different ways. Here, we define transfer learning as any method that transfers knowledge from one machine learning model to another machine learning model. A popular transfer learning technique applied to all kinds of neural networks is pretraining-finetuning, shown in Figure 9A. In this approach, a neural network is first trained by a large inexpensive low-fidelity dataset in the pretraining step. This results in a machine learning model that can predict the target property, but at a lower level of accuracy because of the low-fidelity data. Thereafter, in the finetuning step, the model is retrained by a smaller amount of high-fidelity data, to improve the accuracy of the model. In this finetuning, the parameters of the earlier trained model are used as initial values of the parameters in the optimization procedure. In this way, the pretrained model requires less high-fidelity data as it already has grasped some essential knowledge from the inexpensive low-fidelity data. If the model were trained on the small dataset only, the model would probably get overfitted due to the high number of parameters combined with the low amount of data. As a result, an accurate machine learning model can be obtained even without sufficient high-fidelity data. This technique has already been applied several times to predict thermodynamic and kinetic properties. Ureel et al. (2023b) trained machine learning models to predict the enthalpy of formation, standard entropy, and heat capacity of species belonging to different molecule classes. Using group additivity, they created a large, less accurate, dataset for the pretraining step. The finetuning step was performed using a smaller high-fidelity quantum chemical dataset. This transfer learning approach performed better than training on the small dataset only, but also improved upon the group additivity model, despite using the same quantum chemical data. This thus shows that, using this transfer learning approach, a machine learning model can outperform the classical group additivity approach, even for a small dataset. Also solvation free energies have already been predicted using transfer learning (Vermeire and Green 2021). Here, the larger low-fidelity dataset was a quantum chemical dataset, while the smaller more accurate dataset contained experimental values. This pretraining-finetuning approach has also been applied to the prediction of activation energies in multiple works (Grambow et al. 2020a; Heid and Green 2022; Spiekermann et al. 2022b). In these works, both the pretraining and finetuning steps contained quantum chemically calculated data. The difference between the two datasets is that the finetuning data is calculated at a higher level of theory. Furthermore, Chung and Green (2024) also used transfer learning to predict the solvent correction on gas-phase reaction rates. In this work, the pretraining dataset contains a high amount of uncommon reactions, while the finetuning dataset is smaller, but contains more common reactions. Grambow et al. (2019) also used a transfer learning approach to predict thermodynamic properties but in a slightly different manner. Here, a GNN was trained via transfer learning. Different from the previously stated works, only the output function of the GNN was finetuned. The other parameters, i.e., the parameters in the initialization and iterative step, were kept fixed after pretraining. A quite similar approach was taken by Al Ibrahim and Farooq (2022). In this work an MPNN, followed by an FNN was pretrained on the QM9 dataset. The learned latent molecular representation in the MPNN, combined with the ECFP fingerprint of the molecule, was then used as input to another FNN that predicts the reaction rate of reactions containing this molecule. Here, the FNN of the second model predicting reaction rates was thus trained from scratch, without any pretraining. This is in contrast to the aforementioned method used by Grambow et al. (2019). In that approach, the complete model was thus pretrained, while only a part was finetuned. The opposite technique, namely only pretraining a part of the model, is very popular in language models. As a reminder, these language models usually consist of a BERT encoder, which transforms the input string into a mathematical vector, and an FNN, which transforms that vector into a prediction of the target property. Specific NLP techniques allow to train the BERT encoder solely after which the BERT encoder and the FNN can be jointly finetuned. A major advantage of the approach is that pretraining the encoder does not require labeled data, i.e., molecules or reactions linked with their target property. As a result, solely SMILES or reaction SMILES strings are sufficient to pretrain the model. How this pretraining step is performed is outside the scope of this review, but can be found in the following works that used this technique (Schwaller et al. 2021a; Wang et al. 2019; Zhang et al. 2021). It is also possible to freeze the BERT encoder’s parameters and only optimize the subsequent FNN in the finetuning step. In fact, this completely separates the two steps: the pretraining step creates a vector representation of the molecule/reaction using the BERT encoder, and the finetuning step trains an FNN to transform that vector to the target property. Since the second step is now independent of the encoder, any machine learning model that transforms a vector to the target output can be used (e.g. KRR, SVR, FNN). Another advantage of this independence is that after the pretraining step (training of the encoder), the molecular/reaction representation vector can be used ‘as is’ for many prediction tasks, without the need to train the encoder again. This has been employed multiple times for the prediction of reaction properties. Schwaller et al. (2021a) trained a BERT encoder to obtain a vector representation of the reaction named *rxnfp*. This fingerprint has then been used to create a variety of machine learning models in other works (Griffiths et al. 2024; Heid and Green 2022; Probst et al. 2022).

Above, transfer learning was presented as a method to build a machine learning model using two datasets, usually a less and a more accurate one. Another way to treat two different data qualities is delta-machine learning, shown in Figure 9B. In delta-machine learning, two datasets are required: one dataset containing properties calculated at a fast but less accurate method, and one with properties calculated at a slower but more accurate method. As opposed to transfer learning, it is important that both datasets contain the same molecules or reactions. In delta-learning, instead of training a model to predict the high-fidelity data point, a model is trained to predict the difference between the high-fidelity and low-fidelity data points. By doing so, the machine learning model only needs to predict contributions not included in the cheaper less accurate model. This approach has been employed multiple times to predict the difference between quantum chemically calculated properties and experimentally measured properties (Hu et al. 2003; Meng et al. 2023; Weinreich et al. 2021). Another task for which this has been employed is to predict the difference between a low level of theory and a high level of theory quantum chemically calculated properties (Bogojeski et al. 2020; Ramakrishnan et al. 2015; Ruth et al. 2022). For example, predicting the difference between the enthalpy of formation calculated at G3MP2B3, and the formation enthalpy calculated at B3LYP (Plehiers et al. 2021). The downside of this approach is that B3LYP calculations are still required to obtain an estimate of the G3MP2B3 calculated value. This B3LYP method is faster than the G3MP2B3, but still takes a significant amount of time, and is therefore not ideal for kinetic modeling purposes. This can be mitigated by, instead of using the still time-consuming low-level DFT method as a starting point, using a semi-empirical method such as PM7 or GFN2-xTB (Ramakrishnan et al. 2015; Zhao et al. 2023a).

## 6 Performance assessment of machine learning models

In the previous sections, we have discussed machine learning models according to three pillars: data, representation, and model. Machine learning approaches from separate works usually differ in more than one category, especially if a detailed look is taken at the data, representation, and model. For example, if the same dataset is used as a basis, but cleaned in a different way, it can make the comparison between approaches less objective. Furthermore, even if the same dataset is used for two different machine learning models, the data could be split differently between the training and test subsets, resulting in differing performances. Also, comparing the different representations is difficult. The results obtained when using vector representation of molecules or reactions strongly depend on the expertise of the user selecting the features. Also for graph representation, it is dependent on the features assigned to the atoms and bonds. Another hidden difference is the hyperparameter choice. Most machine learning models require the choice of hyperparameters. A change in these hyperparameters can lead to a significant difference in performance. Thus, even when exactly the same data, data splits, representation, and type of model architecture are used, different outcomes can be expected, due to a difference in hyperparameters. This high number of degrees of freedom makes a comparison between the performances of different machine learning approaches often unreliable. Therefore, here, we will only look at the performances achieved for different tasks, rather than making a comparison between different approaches. For energies, predictions are often assumed to be chemically accurate if the error is smaller than 4.184 kJ/mol (Miller et al. 2021; Ruscic 2014). At a temperature of 450 °C, this error on the Gibbs free activation energy leads to a factor 2 error on the reaction rate constant. This is assumed to be an acceptable error for applications in a kinetic model. However, at room temperature, this error corresponds to a factor 5 on the reaction rate constant. Furthermore, the way researchers define ‘the error’ can vary. Traditionally, the accuracy of thermochemical properties has been described using the width of the 95 % confidence interval. Others, mainly in the field of machine learning, rather use the mean absolute error (MAE) or the root mean square error (RMSE). Evaluating the chemical accuracy of models based on different accuracy metrics can be ambiguous. Requiring the width of the 95 % confidence interval to be lower than 4.184 kJ/mol is usually stricter than requiring the MAE or the RMSE to be lower than this value. Ruscic (2014) noted that using the MAE as metric can underestimate the uncertainty by a factor of 2.5–3.5 in comparison with the width of the 95 % confidence interval. Lastly, it is important to note that the conventional value of 4.184 kJ/mol is rather chosen arbitrarily. It is by no means an important limit below which models suddenly become accurate. Although this value is rather arbitrary and the definition of ‘the error’ can vary, this will be used as a reference to evaluate the performance of machine learning models in what follows. It is important to consider that the reported accuracies are with respect to the test dataset. To obtain an evaluation of the total error on the prediction, the accuracy of the test set data must be taken into account. For example, as mentioned before, the QM9 dataset has a 20 kJ/mol deviation from values calculated at the G4 level of theory. Predicting the energies in this dataset with an error lower that 4.184 kJ/mol would therefore not mean that the obtained thermochemical properties are chemically accurate.

The most basic task is the prediction of gas-phase properties. The most used dataset to train models to predict molecular gas-phase energies is QM9. Different works have achieved energy MAE close to chemical accuracy using this dataset (Faber et al. 2017; Pinheiro et al. 2020, 2022). These works used both the vector and graph representation of the molecules and a wide variety of machine learning models. This QM9 is a high variety dataset, also containing fewer occurring species, which may make the task more difficult. However, when only a subset of QM9 is considered, including only a certain type of molecules, predictions within chemical accuracy can be reached (Dobbelaere et al. 2021a). This was achieved by combining the HDAD representation with an FNN. In the same work, also other thermochemical properties were predicted for a smaller, but more accurate, dataset. The enthalpy of formation, standard entropy, and heat capacity were predicted with MAEs of 9.34 kJ/mol, 3.86 J/mol/K, and 1.47 J/mol/K respectively. For temperatures below 800 °C, this accuracy on the entropy corresponds to errors on energies lower than the chemical accuracy of 4.184 kJ/mol. The error on the enthalpy of formation is rather large, while the same model reached chemical accuracy on a subset of QM9. This is probably due to the smaller dataset size, which makes the model more prone to overfitting.

For the prediction of free solvation energies, RMSE of around 6 kJ/mol can be achieved on the FreeSolv database via the chemistry machine learning package MoleculeNet, using a graph representation (Wu et al. 2018). Similar accuracies are achieved via the open-source chemical machine learning package Chemprop, also using a graph representation (Heid et al. 2024; Yang et al. 2019). However, this dataset is not a good benchmark to evaluate the prediction of solvation energies, since it only includes water as a solvent. Nonetheless, different studies predicting the solvation energies using suitable datasets (containing a variety of solutes and solvents) show results within chemical accuracy (Chung et al. 2022; Liao et al. 2023a; Pathak et al. 2020; Subramanian et al. 2020; Vermeire and Green 2021). These works again used both vector and graph representations combined with different models. This might, however, still not give an objective representation of the performance. Often, when test data is fed to the model, the model has already seen both the solvent and the solute during training, only not together. These test data points thus do not show an objective evaluation of how the model would perform when being fed a new solute or solvent. Vermeire and Green (2021) tested how their machine learning model (GNN) would perform on solvents it had not seen during training. For almost all tested solvents, the RMSE was still within the limits of chemical accuracy.

For the prediction of adsorption energies, the models using quantum chemical descriptors, such as the d-band center, are irrelevant, since they are, due to their high computational cost, not suited for kinetic modeling purposes. If these d-band features are calculated in a faster manner, this approach is interesting to consider. Noh et al. (2018) showed that this faster approach could predict adsorption energies of CO on a (100) facet with an RMSE of 17 kJ/mol, using a vector representation and KRR as model. When the training dataset was extended via active learning, an error of only 5 kJ/mol was achieved. This, however, only considered one adsorbate. In another work, a GNN created to predict the adsorption energy of several adsorbates on several catalysts yielded an MAE of around 17 kJ/mol, while the accuracy on DFT data used for training and testing was of the same order of magnitude (Pablo-García et al. 2023). It is thus unclear whether the error is due to the shortcomings of the machine learning models, or the error on the training and test data. In the field of predicting kinetics with machine learning, the focus has mainly been on the prediction of the activation energy. For a dataset containing a high variety of DFT-calculated gas-phase reactions, an MAE of around 18 kJ/mol was reached, using a GNN (Heid and Green 2022). This large error is at least partially due to the variety of the dataset. On another, smaller but more reaction-specific dataset, the same model namely yielded an MAE of 11 kJ/mol. Approximately the same accuracies were reached for the same dataset in other works (Heinen et al. 2021; Stuyver and Coley 2022). Lastly, models have also been trained to predict the free activation energy of liquid-phase reactions, more specifically nucleophilic aromatic substitutions. Models trained and tested on this dataset show MAEs within chemical accuracy (Heid and Green 2022; Jorner et al. 2021), both when using a vector and graph representation. Although this is partially due to the small range of activation energies in the dataset, it is a very promising result showing the potential of the prediction of the rate of relevant reactions.

Transfer learning approaches usually yield better results than training a model with a single dataset (Spiekermann et al. 2022b; Vermeire and Green 2021). One approach in particular interesting for kinetic modeling purposes is a transfer learning approach where the pretraining is performed using a GAV database. The advantage of this is that only the same data as was needed to fit GAVs is required. Models trained with this approach showed errors within chemical accuracy, while this accuracy was not reached when predicting the properties with GAVs only. Also, delta-learning yields better results than direct learning (Weinreich et al. 2021). Here, however, it is important for kinetic modeling purposes that the low-level dataset is generated by a fast and automated method. The improvements achieved when using these faster methods with respect to direct prediction have, to our knowledge, not yet been reported.

Overall, machine learning models show promising results to be used for kinetic modeling purposes. For most properties, there are different models that show errors within or close to the limits of chemical accuracy. Worse accuracies are obtained when the model is trained for a wide range of molecules or reactions with a limited training dataset size. Datasets containing only a small part of the molecular or reaction space can usually make accurate predictions, especially when using a transfer learning approach.

## 7 Conclusion and perspective

This review explores the potential of machine learning to predict thermodynamic and kinetic properties, focusing on their integration into detailed kinetic models. Currently, detailed chemical kinetic models rely on methods such as quantum chemistry or rapid approximations like group additivity for property prediction, each with its limitations: quantum chemical calculations are slow and the rapid approximations are often inaccurate. Hence, machine learning presents a promising alternative. We examine the current state-of-the-art in machine learning for property prediction, emphasizing three key pillars: data, representation, and model. Notably, the scarcity of accurate data emerges as the primary obstacle to machine learning’s integration into detailed kinetic models. Accurate data exists but is scarce and scattered around the literature. Larger datasets on the other hand, typically comprise properties that are calculated at a low level of theory.

The representation and model pillars are closely intertwined. Graph representations offer rich chemical information but often require large machine learning models, leading to overfitting when using small datasets. Conversely, vector representations generally contain less detail but are compatible with smaller models. Both types of representations, coupled with various mathematical models, yield promising results across different property prediction tasks. Moreover, models trained on datasets covering only a limited range of the molecular or reaction space consistently yield chemically accurate results in prediction tasks, suggesting that the current state of the representation and model pillars suffices for kinetic modeling.

Although many advances have been made in these two pillars, the main challenge lies in data scarcity. To mitigate this, more data-efficient training techniques are needed. Transfer learning, for instance, leverages a larger low-fidelity dataset to aid training on a small high-fidelity dataset, reducing overfitting. This method has demonstrated its efficacy across various prediction tasks, even when utilizing low-fidelity data generated through group additive values.

Delta-learning, on the other hand, trains models to predict differences between low- and high-fidelity calculated or experimentally determined properties. However, its application in detailed kinetic models requires advancements in semi-empirical techniques for efficient low-level calculations to speed up the prediction process.

Overall, while progress has been made in developing machine learning models and representations, overcoming data scarcity remains crucial for their effective application in detailed kinetic models. Addressing this challenge through the creation of larger, more accurate datasets and the development of data-efficient machine learning techniques holds promise for enhancing the capabilities of kinetic modeling.
